# Supplementary material for: Tunable backbone-degradable robust tissue adhesives via in situ radical ring-opening polymerization
Source: Nat Commun. 2023 Sep 28;14:6063. doi: 10.1038/s41467-023-41610-1 (PMC10539349; doi:10.1038/s41467-023-41610-1)
Supplement: Supplementary file 1 — Supplementary information [file 41467_2023_41610_MOESM1_ESM.pdf]

# Supplementary Information

# Tunable backbone-degradable robust tissue adhesives via in situ radical ring-opening polymerization.

Ran Yang[1,2], Xu Zhang[1], Binggang Chen[1*], Qiuyan Yan[1*], Jinghua Yin[1], Shifang Luan[1,2*]

Correspondence to: bgchen@ciac.ac.cn; qyyan@ciac.ac.cn; sfluan@ciac.ac.cn

Requests for materials should be addressed to B.C. (bgchen@ciac.ac.cn)

**This PDF file includes:**

Supplementary Figs 1 to 30

Supplementary Tables 1 to 2

**Other Supplementary Materials for this manuscript include the following:**

Supplementary Movie 1 to 5

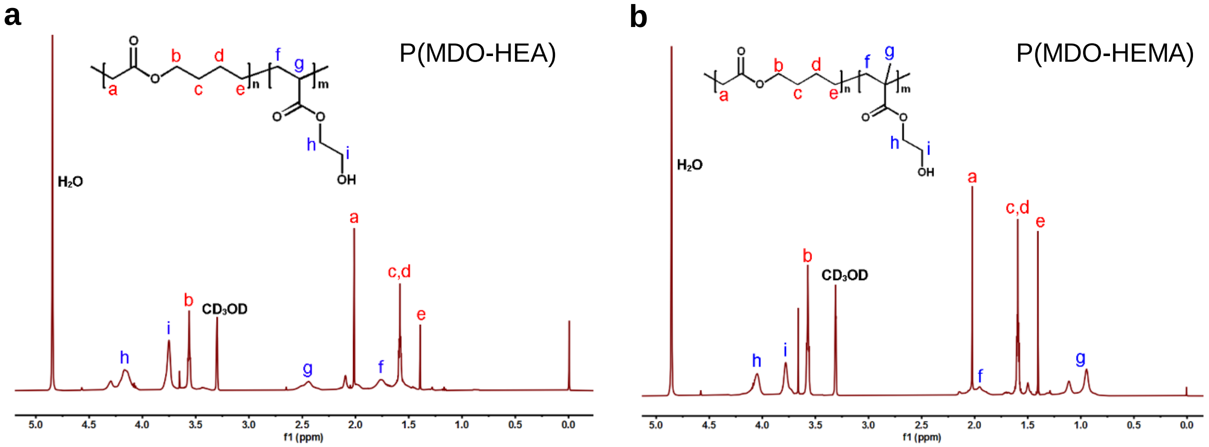


c

| Sample name | Feed Composition MDO:HEMA | Copolymer Composition[a] MDO: HEMA | Mn[b] (Da) | Mw/Mn |
|---|---|---|---|---|
| $MDO_1$-$HEMA_1$ | 1:1 | 0.77:1 | 20187 | 2.84 |
| $MDO_1$-$HEMA_2$ | 1:2 | 1.10:2 | 11874 | 2.40 |
| $MDO_1$-$HEMA_3$ | 1:3 | 0.90:3 | 11438 | 2.39 |
| $MDO_1$-$HEMA_4$ | 1:4 | 0.98:4 | 12312 | 2.29 |
| $MDO_1$-$HEMA_5$ | 1:5 | 0.95:5 | 14017 | 2.33 |

[a] As determined from $^1$HNMR [b] As measured by GPC in DMF

**Supplementary Fig. 1 | Characterization of the chemical structure of the BDRAs. a**, **b**, Proton nuclear magnetic resonance ($^1$H-NMR) spectroscopy of BDRA ($MDO_1$-$HEA_1$) (**a**) and BDRA ($MDO_1$-$HEMA_1$) (**b**) in $CD_3OD$. **c**, Molar ratio of MDO to HEMA in the initial monomer feed and the purified copolymer determined by $^1$H NMR. The number average molecular weight (Mn) and distribution (Mw/Mn) were acquired by GPC.

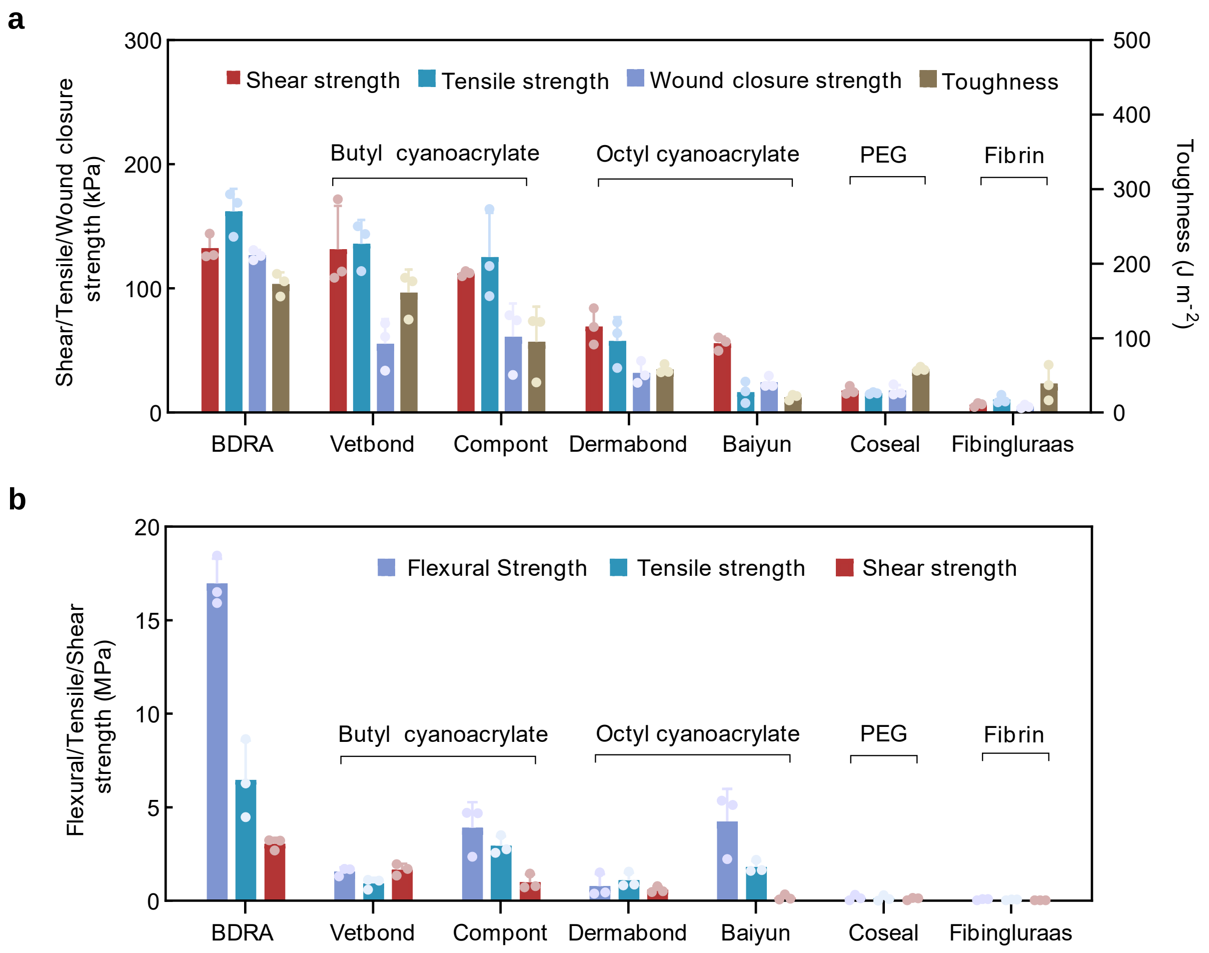


**Supplementary Fig. 2 | Comparison of wet tissue adhesion properties between the BDRAs and various commercially available tissue adhesives. a,** Shear, tensile and wound closure adhesion strength, as well as adhesion toughness for porcine skin. **b,** Flexural, tensile, and shear adhesion strength for bovine bone. Data of **(a-b)** are presented as the means ± SDs, $n$ = 3 independent samples.

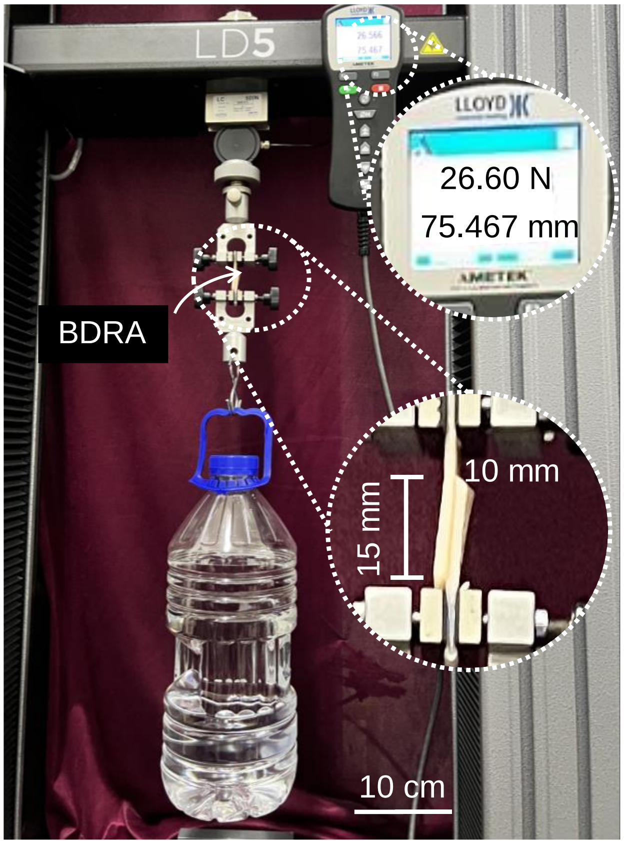


**Supplementary Fig. 3 |** The high bearing capacity of bonded porcine skin (Area: 10 × 15 $mm^2$) using the BDRA ($MDO_1$-$HEA_1$-$NHS_{1/2}$).

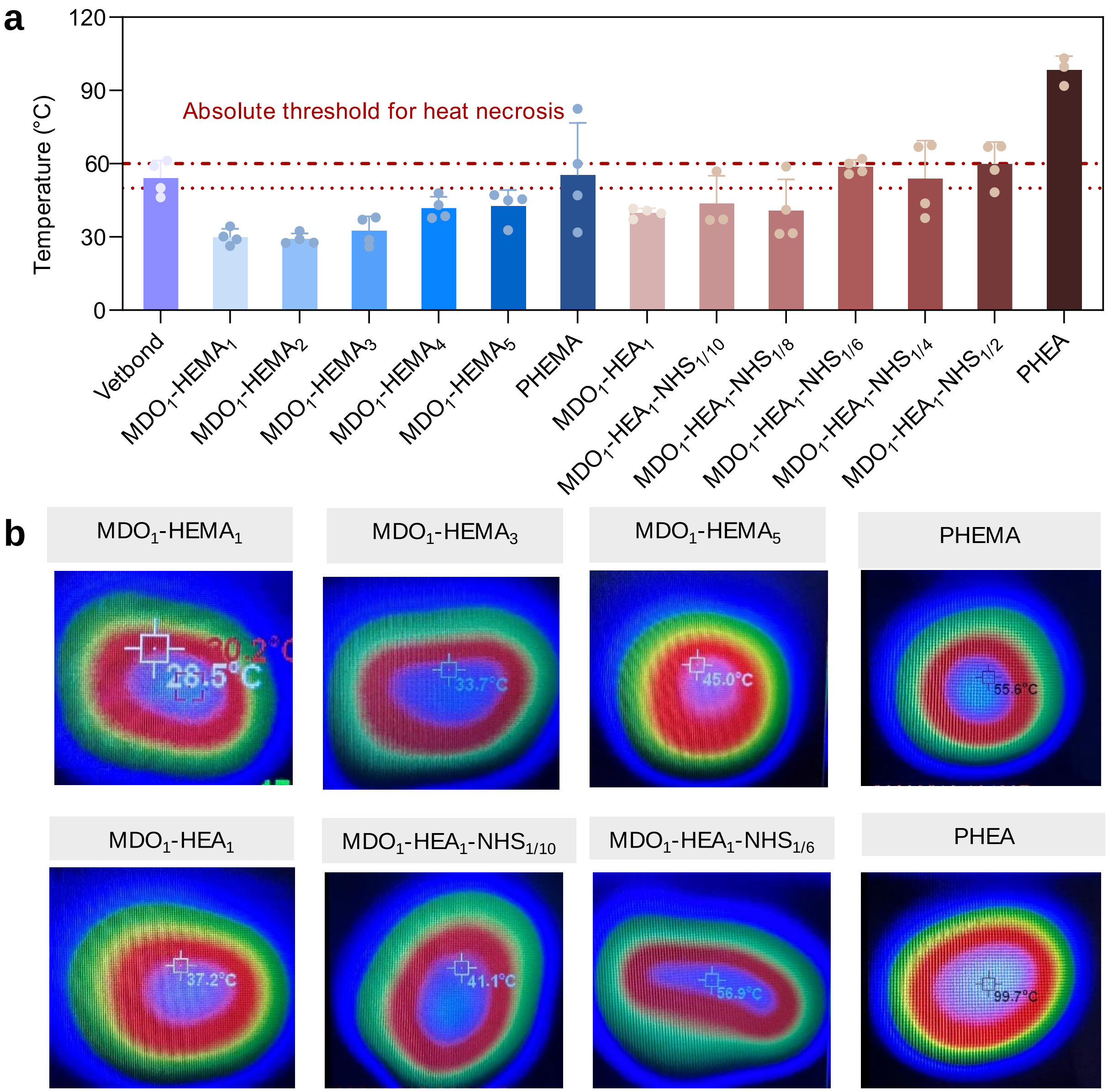


**Supplementary Fig. 4 | Thermal effects of BDRAs on porcine skin. a,** The peak temperature of BDRAs and Vetbond and acrylate homopolymer system (PHEA and PHEMA) by applying 20 μL of adhesive to an area of 1cm × 1cm on the surface of the porcine skin. Data are presented as means ± SDs, *n* = 3 independent samples in PHEA and $MDO_1$-$HEA_1$, and *n* = 4 independent sample for others. **b,** Representative thermal images of the highest temperature on the surface of pigskin captured by a thermal imager. Three times each experiment were repeated independently with similar results.

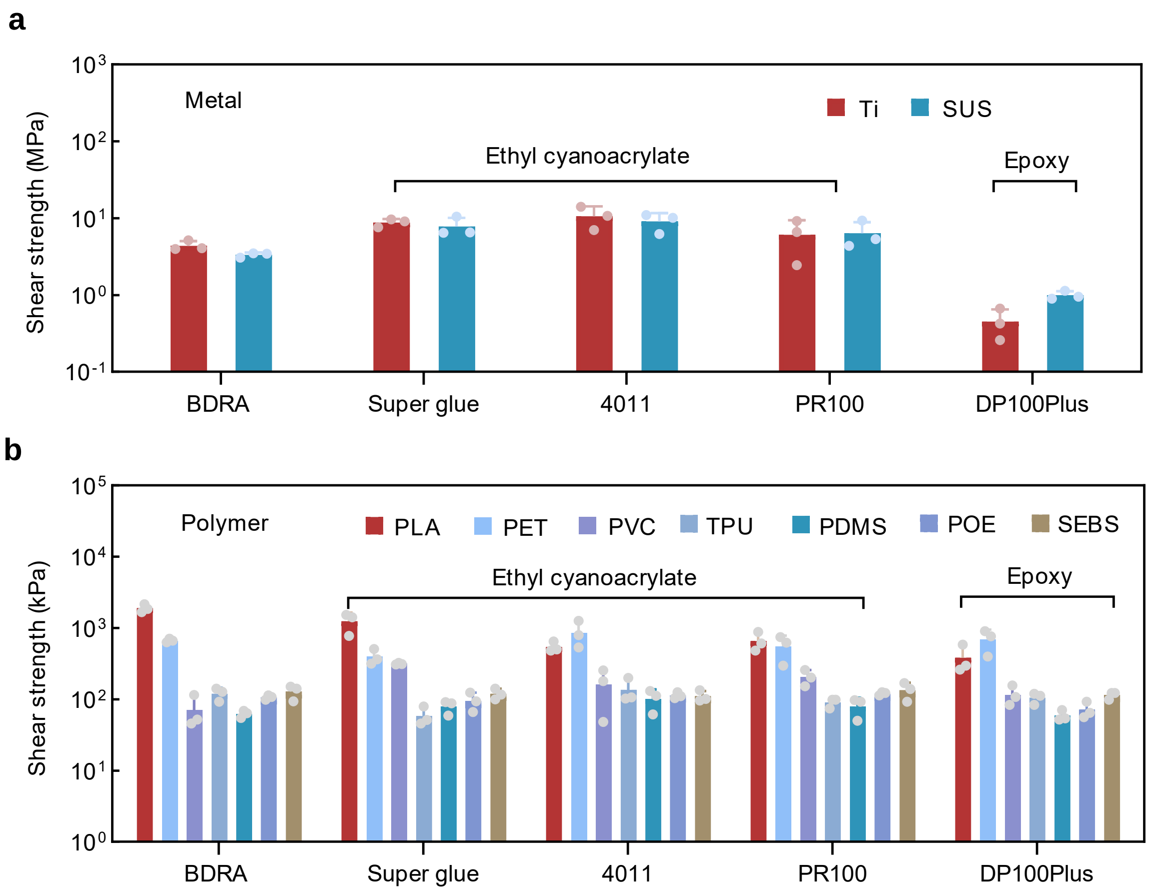


**Supplementary Fig. 5 | Comparison of adhesion properties of the BDRAs and various commercially available engineering adhesives for metals and polymers. a**, Shear adhesion strength for titanium (Ti) and steel use stainless (SUS). Data are presented as the means ± SDs, $n$ = 3 independent samples. **b**, Shear adhesion strength for polylactic acid (PLA), polyethylene glycol terephthalate (PET), polyvinyl chloride (PVC), thermoplastic polyurethanes (TPU), polydimethylsiloxane (PDMS), polyolefin thermoplastic elastomer (POE), and styrene ethylene butylene styrene (SEBS). Data are presented as the means ± SDs, $n$ = 3 independent samples.

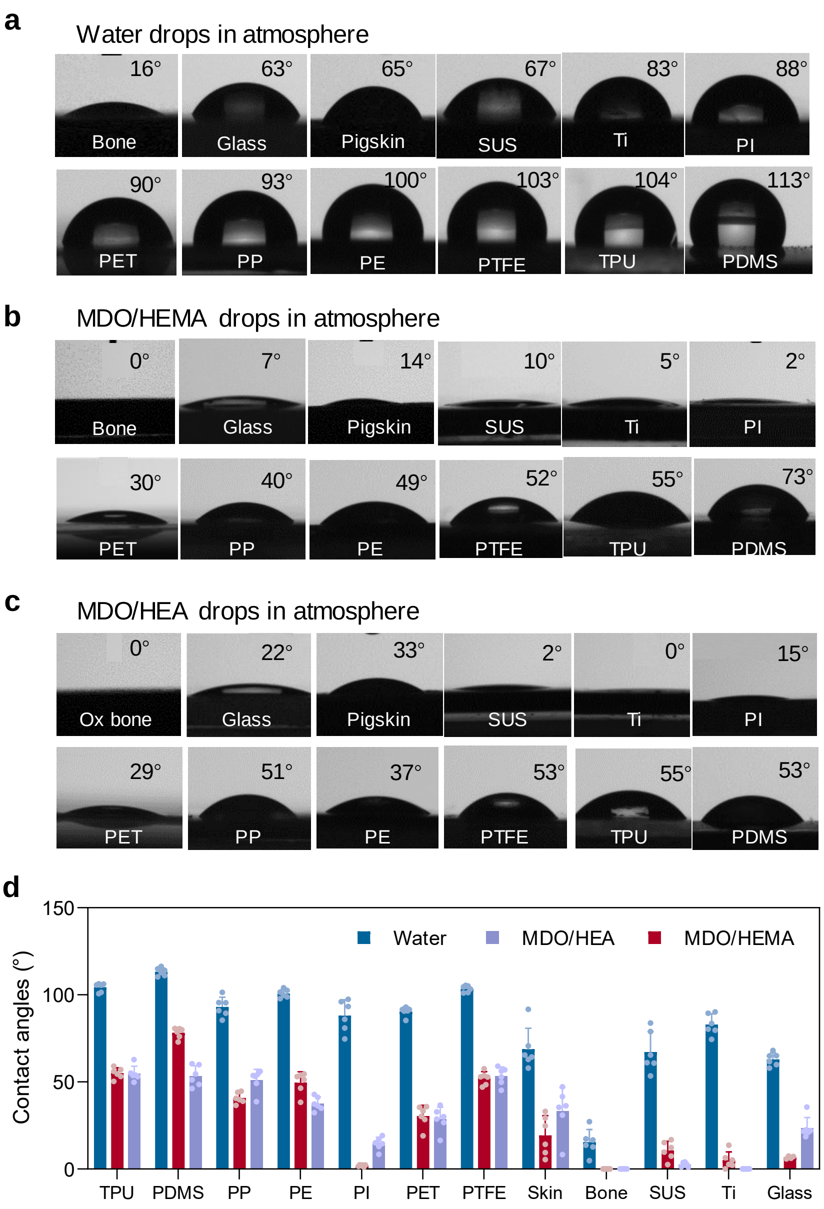


**Supplementary Fig. 6 | The surface wettability of BDRAs to various substrates. a-c**, The contact angles of water droplets (**a**), the BDRA precursor (MDO/HEMA (1:1) mixture without initiator) (**b**), and the BDRA precursor (MDO/HEA (1:1) mixture without initiator) (**c**) for various substrates. **d**, Characterize the contact angle of BDRAs on various substrates. Data are presented as means ± SDs, *n* = 6 independent samples. The substrates include animal tissues (bone, pigskin), inorganic glass, metal (titanium (Ti), steel use stainless (SUS)), and polymers such as polyethylene terephthalate (PET), polypropylene (PP), polyethylene (PE), polytetrafluoroethylene (PTFE), thermoplastic polyurethanes (TPU), polydimethylsiloxane (PDMS), polyimide (PI).

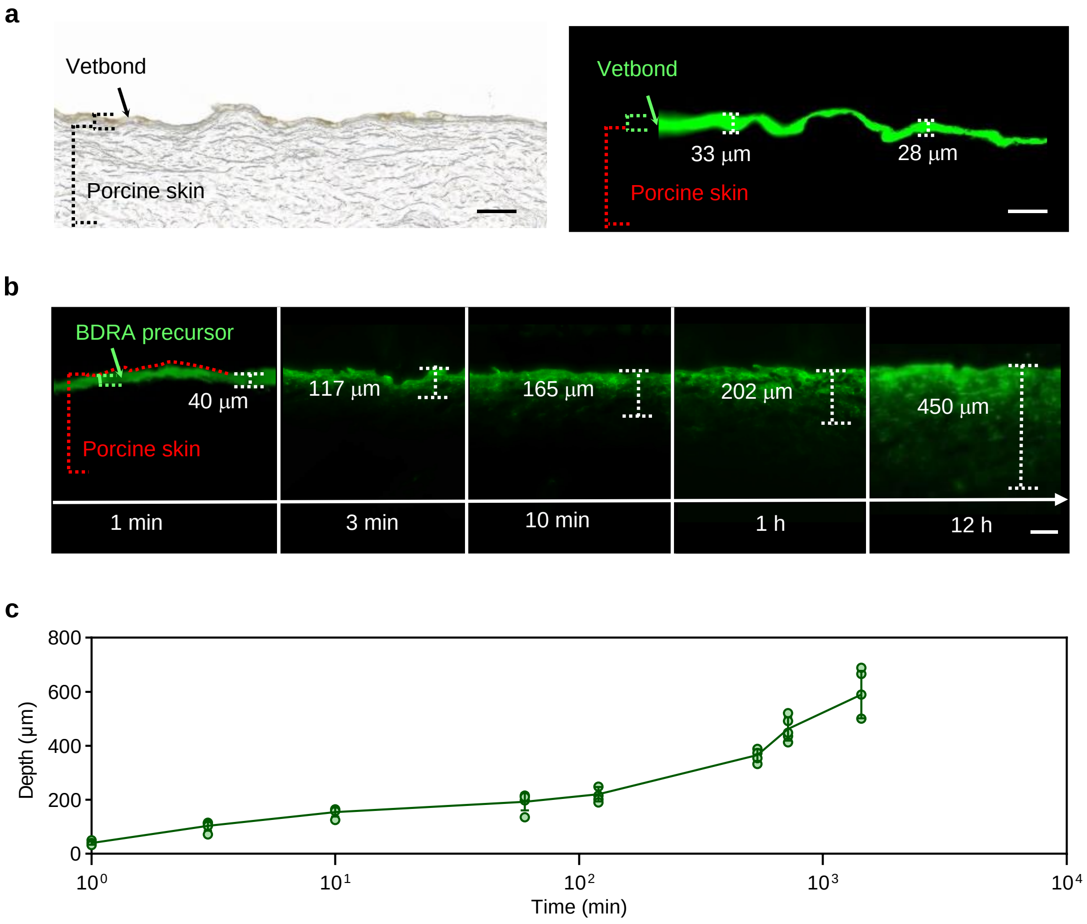


**Supplementary Fig. 7 | Penetration performance of the BDRAs. a**, Bright field image (left) and dark-field fluorescent image (right) of CA (Vetbond) to porcine skin, scale bar = 100 μm. **b**, **c**, The representative fluorescence images (**b**) and penetration depth (**c**) of the BDRA precursor (MDO/HEA (1:1) mixture without initiator) to porcine skin varies with time. Data are presented as means ± SDs for $n$ = 5 independent samples, scale bar = 100 μm. The experiments in (**a-b**) were repeated independently three times with similar results.

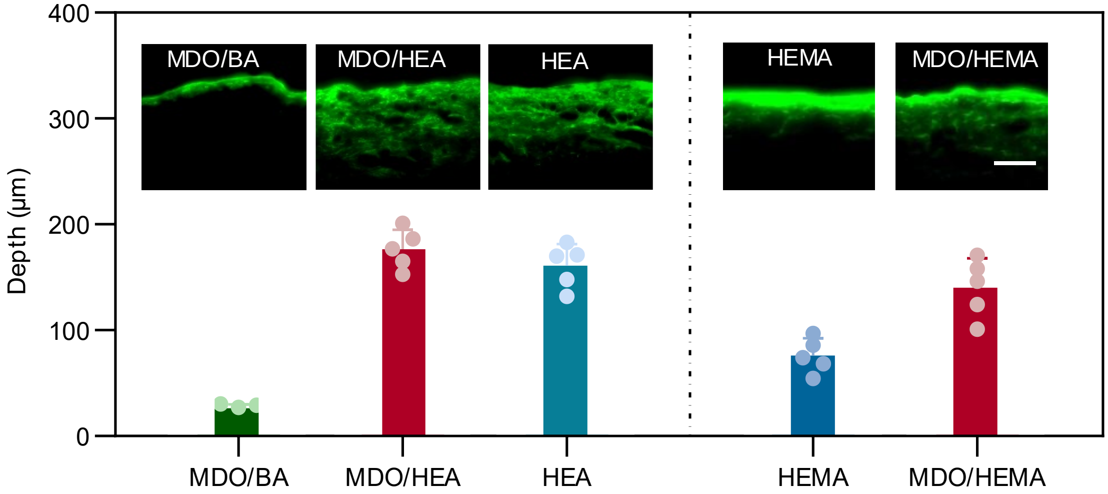


**Supplementary Fig. 8 | Synergistic effect of hydrophilic-hydrophobic composition on the penetration of the BDRAs.** The penetration depth of the precursors (hydrophilic HEA and HEMA, hydrophobic MDO/BA (1:1), and amphiphilic MDO/HEA (1:1) and MDO/HEMA (1:1)) without initiator after 1 hour applied to porcine skin, scale bar = 100 μm. Data are presented as means ± SDs for $n = 5$ independent samples.

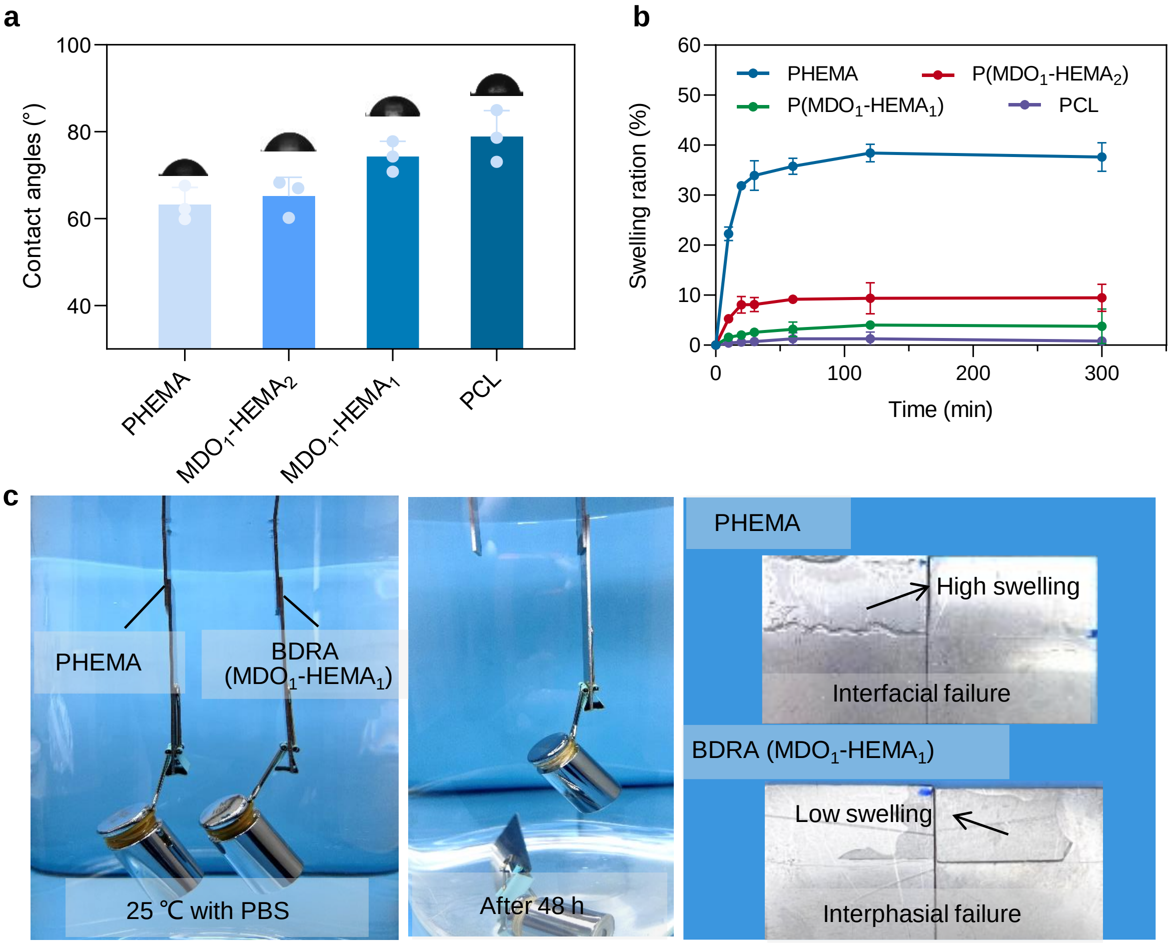


**Supplementary Fig. 9 |The swelling behavior and underwater adhesion stability of the BDRAs. a**, Water contact angles of PCL, PHEMA and the BDRAs (MDO-HEMA). **b**, Swelling behavior of PCL, PHEMA, and the BDRAs (MDO-HEMA) in PBS at 37 °C. **c**, Adhesion durability and failure mode of the BDRA ($MDO_1$-$HEMA_1$) and PHEMA for SUS in PBS at 25 °C. Data of (**a-b**) are presented as means ± SDs for *n* = 3 independent samples.

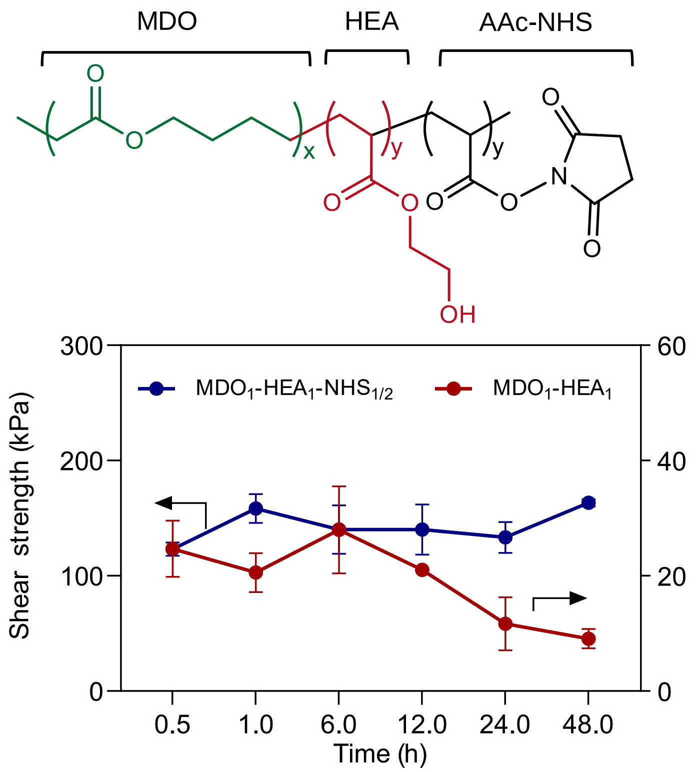


**Supplementary Fig. 10 | Adhesion performance of the NHS-modified BDRA.** Shear adhesion strength between the BDRA ($MDO_1$-$HEA_1$-$NHS_{1/2}$) and wet porcine skins with time. Data are presented as means ± SDs for $n = 3$ independent samples.

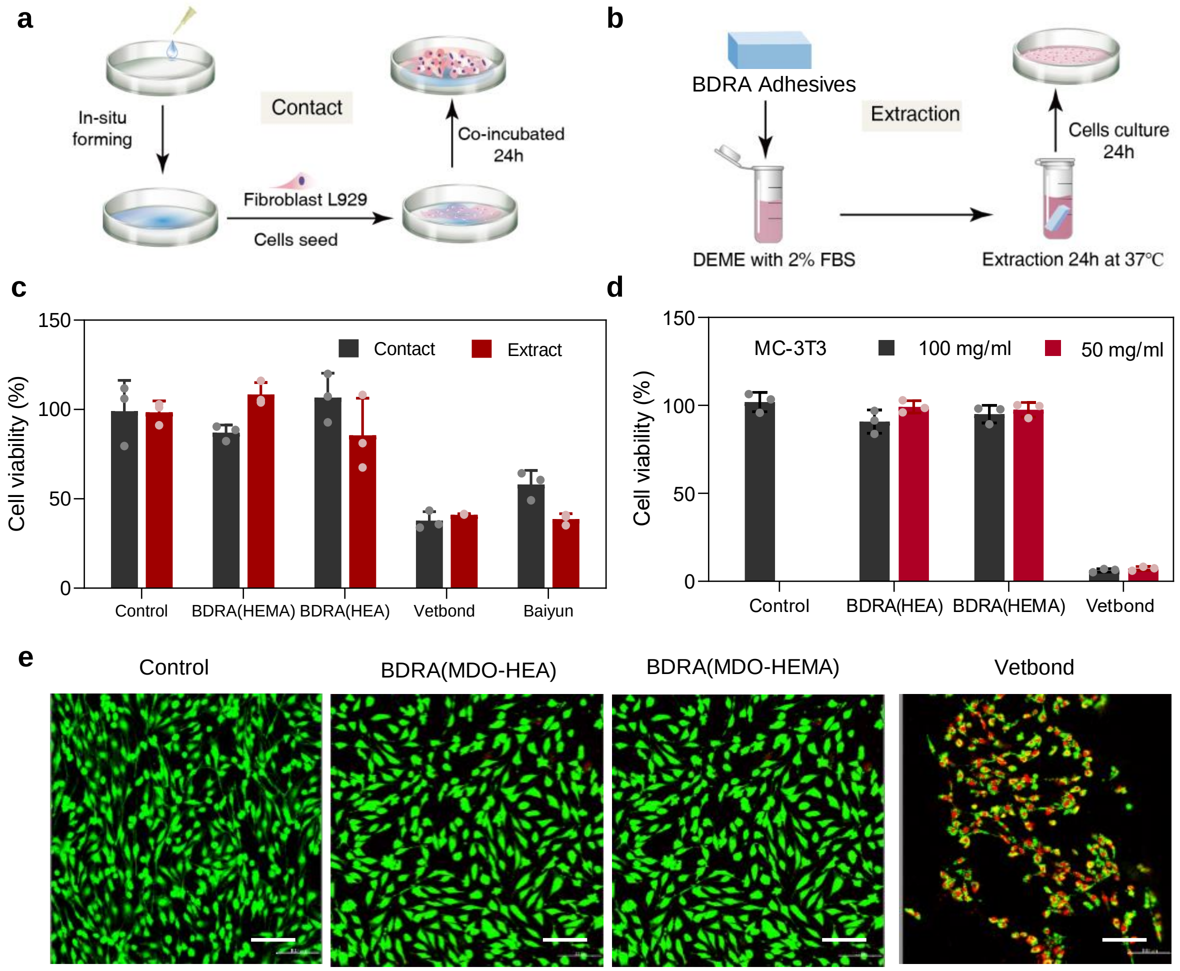


**Supplementary Fig. 11 | Cytocompatibility of the BDRAs in vitro**. **a**, **b**, Schematic of the cytocompatibility tests of the BDRAs via incubation of the L929 cells by direct contact (**a**) and the BDRA's extracts (**b**) for 24 hours. **c**, Cell viability of L929 measured by CCK-8 assay. 2% DEME was taken as control. **d**, Cell viability of MC-3T3 measured by CCK-8 assay. 2% DEME is taken as control. Data of (**c-d**) are presented as means ± SDs for $n$ = 3 independent samples. **e**, Representative confocal images of the MC-3T3 cells within live/dead assay. Scale bar = 100 μm. Three times each experiment were repeated independently with similar results.

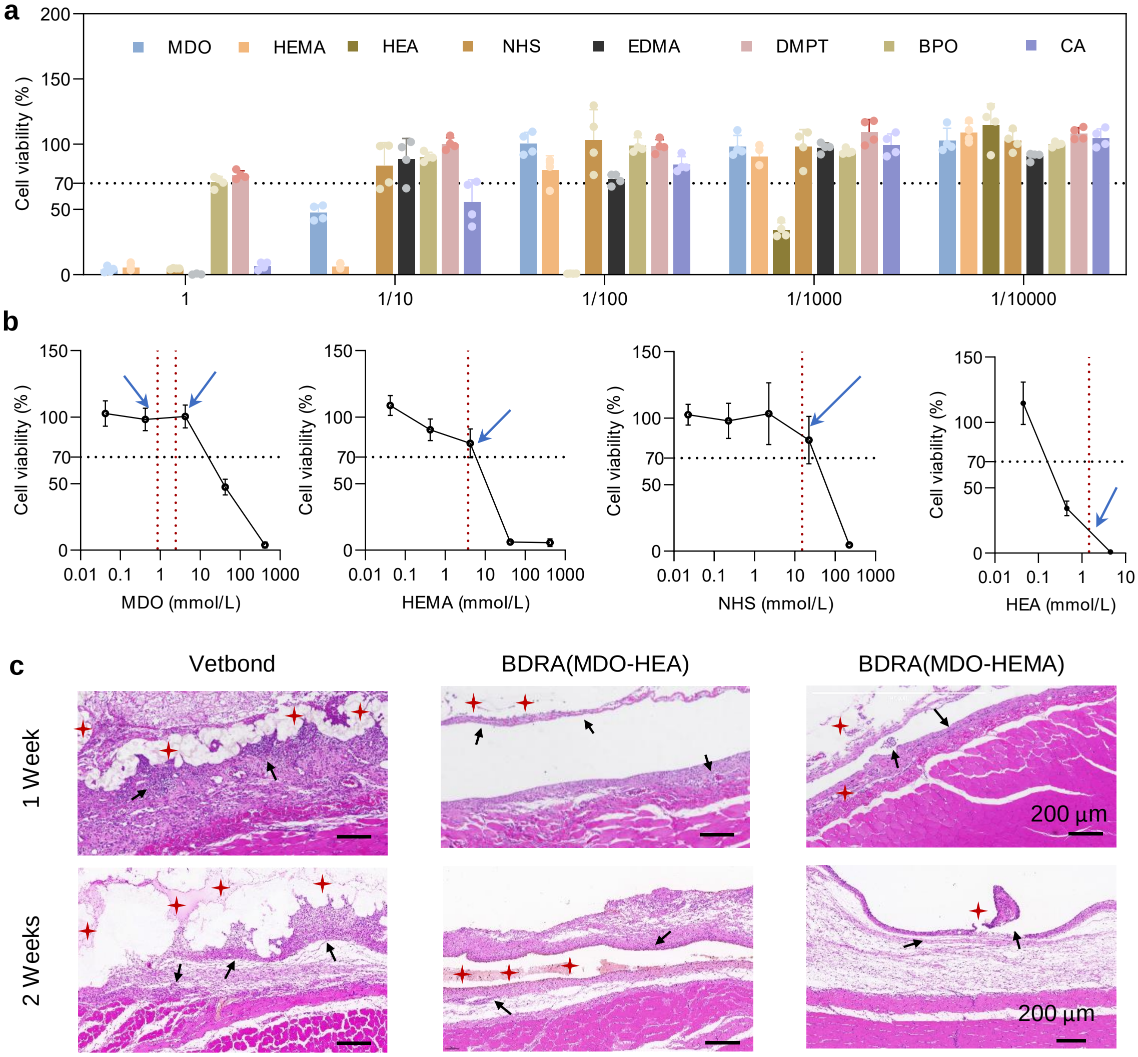


**Supplementary Fig. 12 | Biocompatibility of the residual precursors from BDRAs. a,** Cell viability of L929 co-incubated with involved precursors of BDRAs measured by CCK-8 assay. The theoretical maximum residual concentration of monomer, which does not participate in the reaction and remains complete is represented by 1 and is diluted ten times to 1/10000. CA was taken as control. **b,** The concentrations of eluted residual precursors from BDRA systems and the corresponding cell viability. The highest concentrations of residual MDO, HEMA, HEA, and NHS from adhesive systems were 2.4 mmol/L, 3.72 mmol/L,1.45 mmol/L, and 15.08 mmol/L, respectively. Data of (**a-b**) are presented as means ± SDs for $n$ = 4 independent samples. **c,** Representative H&E staining of abdominal subcutaneous tissues at 1 and 2 weeks after BDRA in-situ curing, scale bar = 200 μm. The red stars represent glues, and the black arrows indicate the indicate the presence of inflammatory cells. Three times each experiment were repeated independently with similar results.

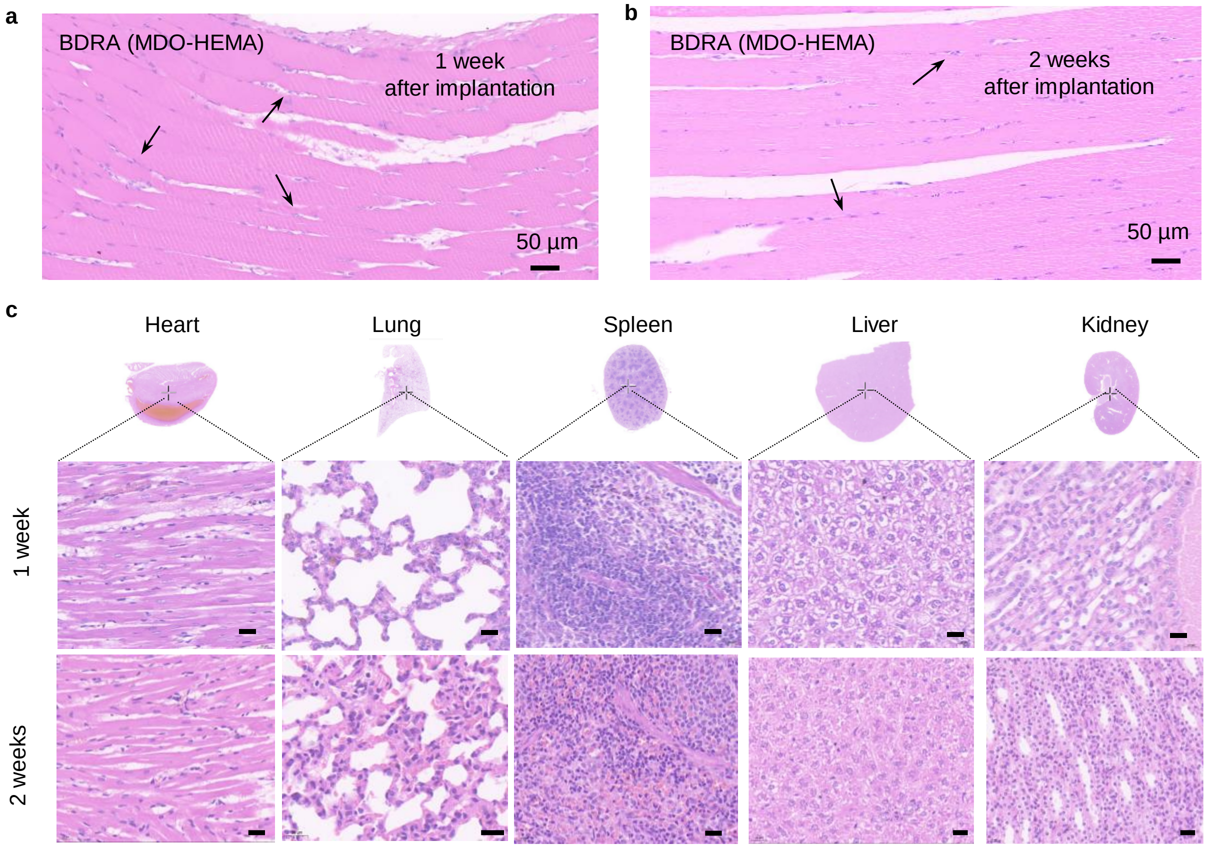


**Supplementary Fig. 13 | Histocompatibility of the BDRAs. a**, **b**, Representative histological images of rat tissues around dorsal implanted BDRA ($MDO_1$-$HEMA_1$) at 1 week (**a**) and 2 weeks (**b**). The black arrow represents inflammatory cells, scale bar = 50 µm. **c**, The representative H&E staining of the heart, liver, spleen, lung, and kidney after the BDRAs implanted for 1 and 2 weeks, scale bar = 20 µm. The experiments in (**a-c**) were repeated independently three times with similar results.

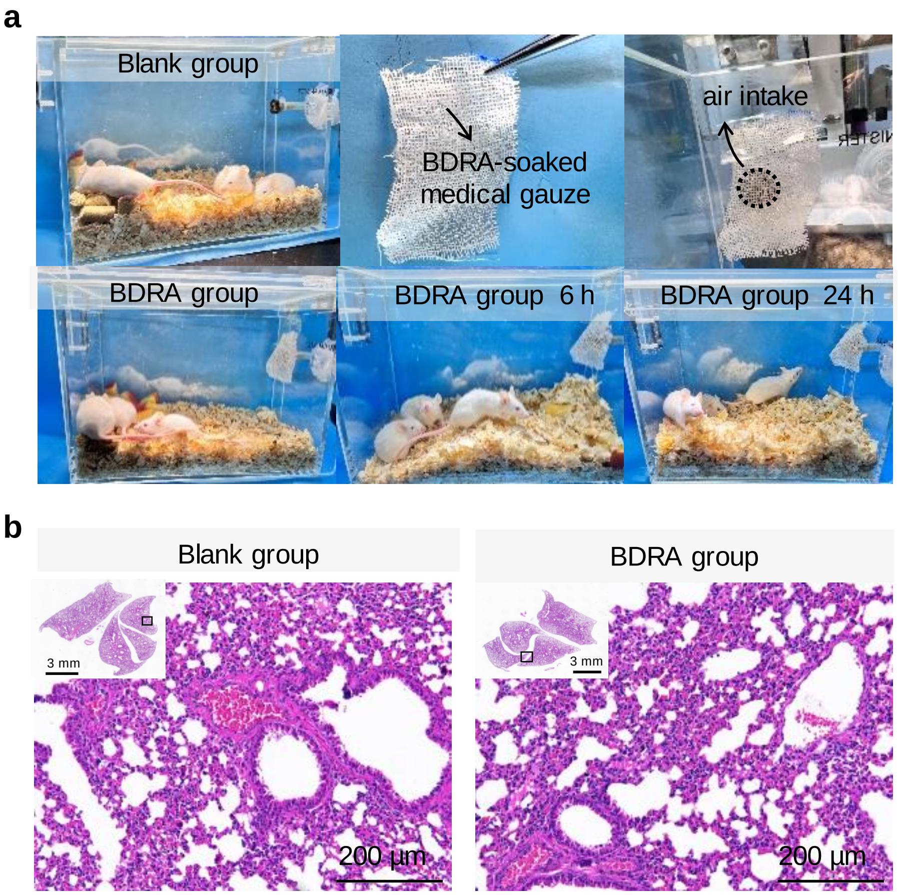


**Supplementary Fig. 14 | Pulmonary toxicity of the BDRAs. a**, Digital photographs of the experiments for evaluating pulmonary toxicity by volatile organic compounds released from the BDRA ($MDO_1$-$HEMA_1$). BALB/c mice were randomly divided into two groups ($n$ = 3). **b**, H&E-stained images of lung tissues after exposure to the BDRAs for 24 hours. Three times each experiment were repeated independently with similar results.

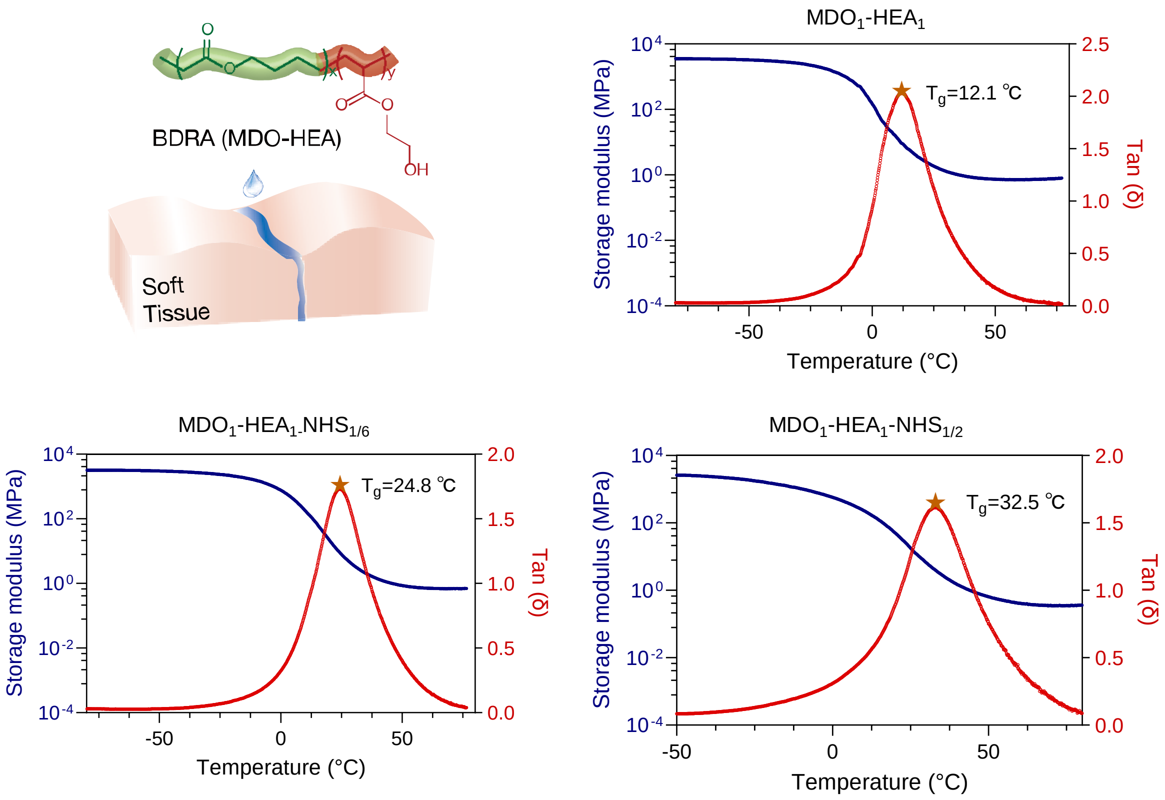


**Supplementary Fig. 15 | The dynamic mechanical analysis of the BDRAs (MDO-HEA).** The storage modulus and tan(δ) of BDRAs with the temperature at a frequency of 1 Hz, and the molar ratio of MDO to HEA was 1:1, and NHS to MDO ranges from 1:0 to 1:1/2.

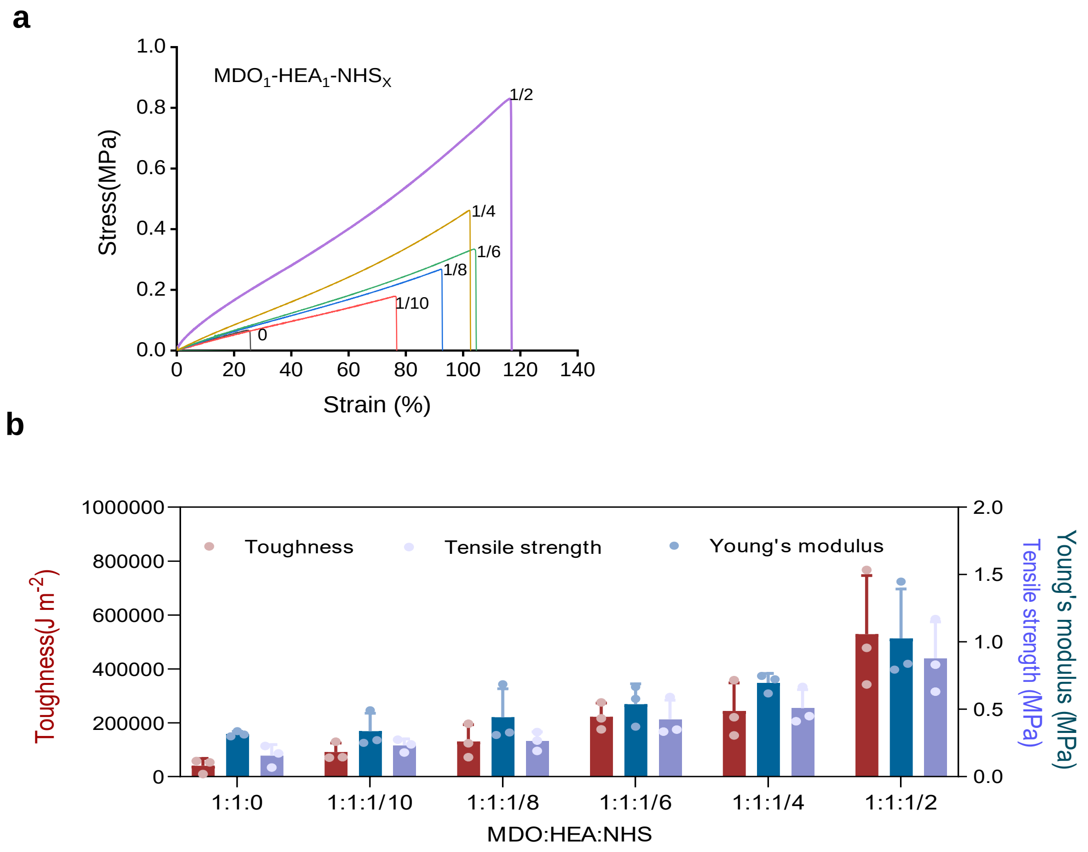


**Supplementary Fig. 16 | The mechanical characterization of the BDRAs (MDO-HEA). a**, Representative tensile curves of the BDRAs. **b**, Mechanical properties of the BDRAs including Young's modulus, tensile strength, elongation at break, and toughness. Data are presented as means ± SDs for *n* = 3 independent samples.

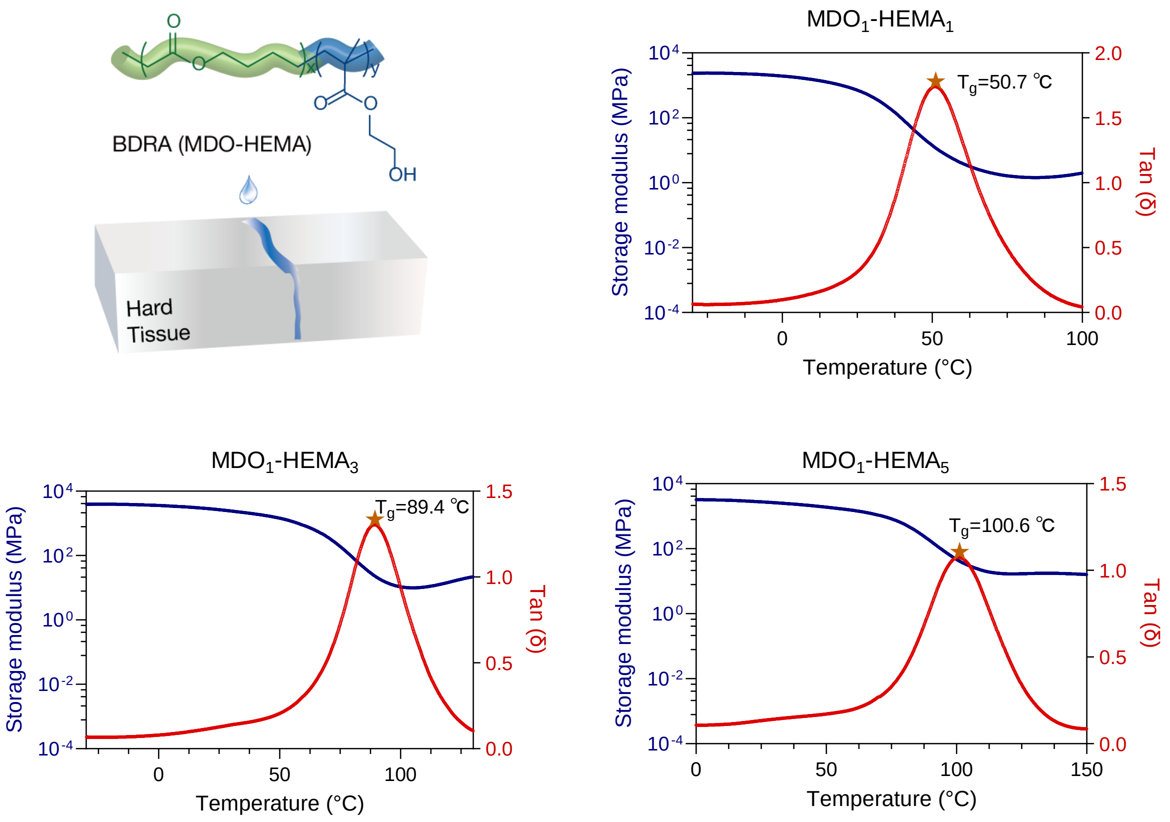


**Supplementary Fig. 17 | The dynamic mechanical analysis of the BDRAs (MDO-HEMA)**. The storage modulus and tan(δ) of BDRAs with temperature at a frequency of 1 Hz, and the molar ratio of MDO to HEMA ranges from 1:1 to 1:5.

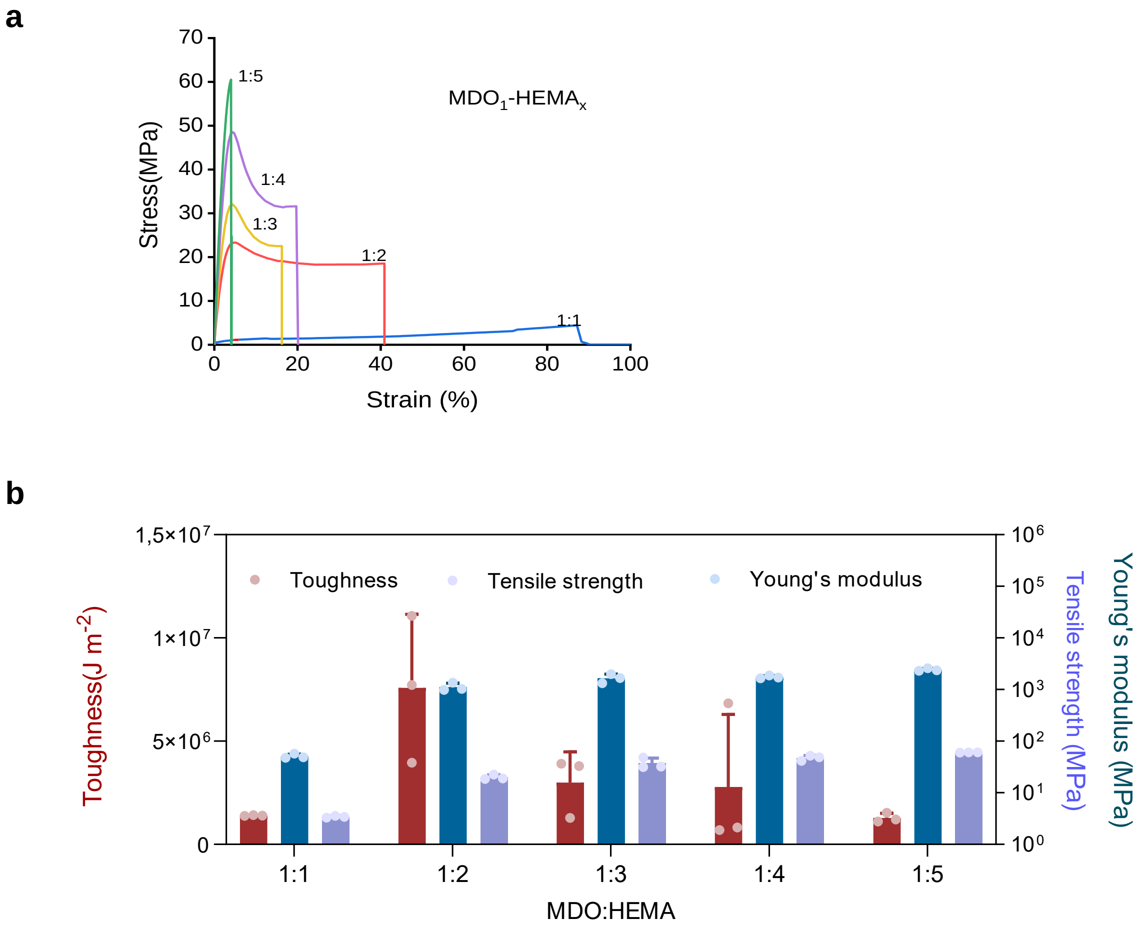


**Supplementary Fig. 18 | The mechanical characterization of the BDRAs (MDO-HEMA). a**, Representative tensile curves of the BDRAs. **b**, Mechanical properties of the BDRAs (MDO-HEMA), including Young's modulus, tensile strength, elongation at break, and toughness. Data are presented as means ± SDs for $n$ = 3 independent samples.

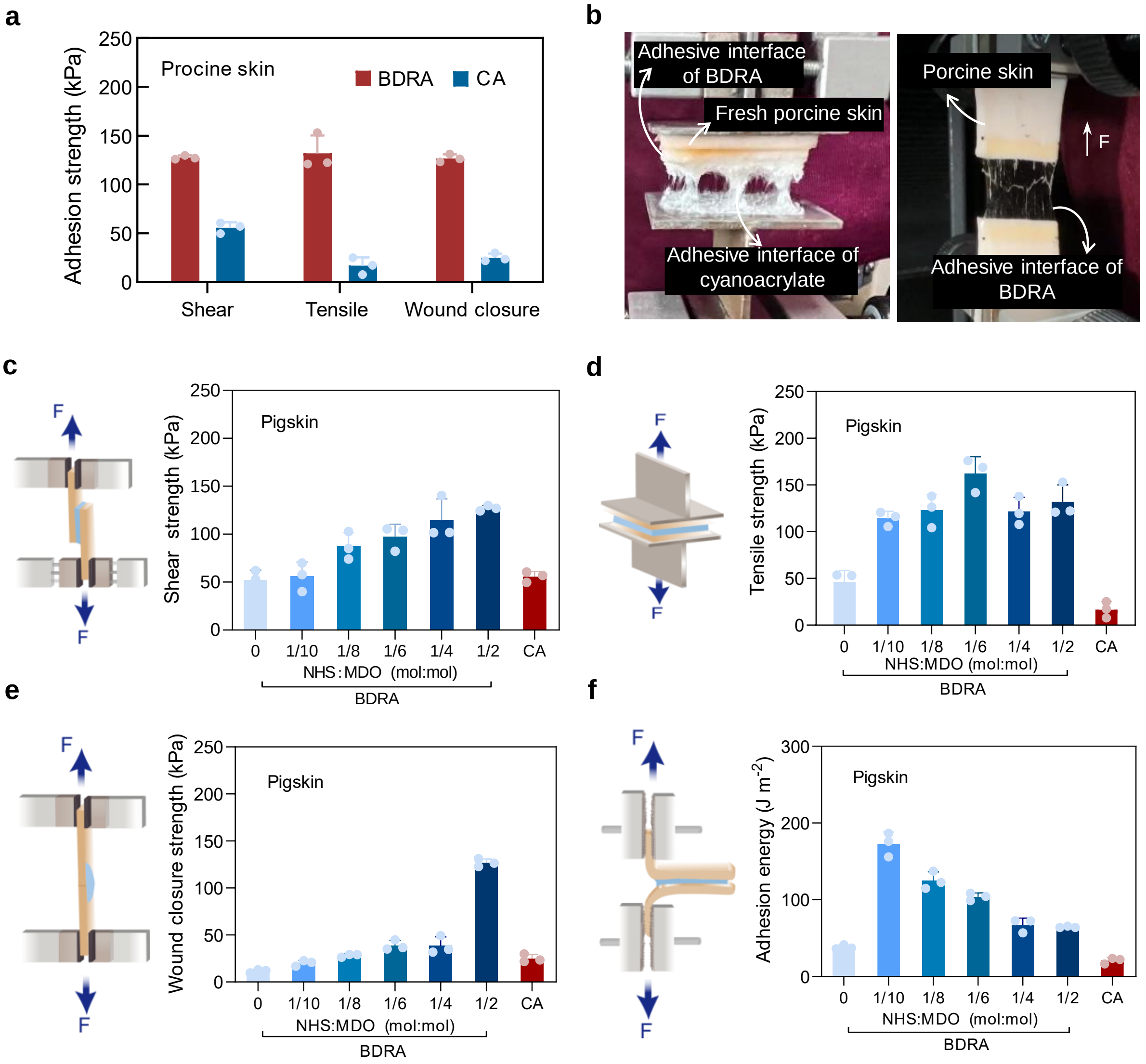


**Supplementary Fig. 19 | The adhesion measurement of the BDRAs to pigskin.** **a**, Adhesion performance of the adaptive BDRA ($MDO_1$-$HEA_1$-$NHS_{1/2}$) for wet soft porcine skin. **b**, Representative photos of wet soft tissue adhered by the BDRAs. **c**, **d**, **e**, **f**, Shear (**c**), tensile (**d**), wound closure adhesion strength (**e**) and adhesion energy (**f**) vary with monomer proportion of the BDRAs (MDO-HEA-NHS), the molar ratio of MDO to HEA was 1:1, and NHS: MDO (mol:mol) was from 0 to 1/2. And octyl cyanoacrylate (Bai yun) as a reference. Data of (**a, c-f**) are presented as means ± SDs for *n* = 3 independent samples.

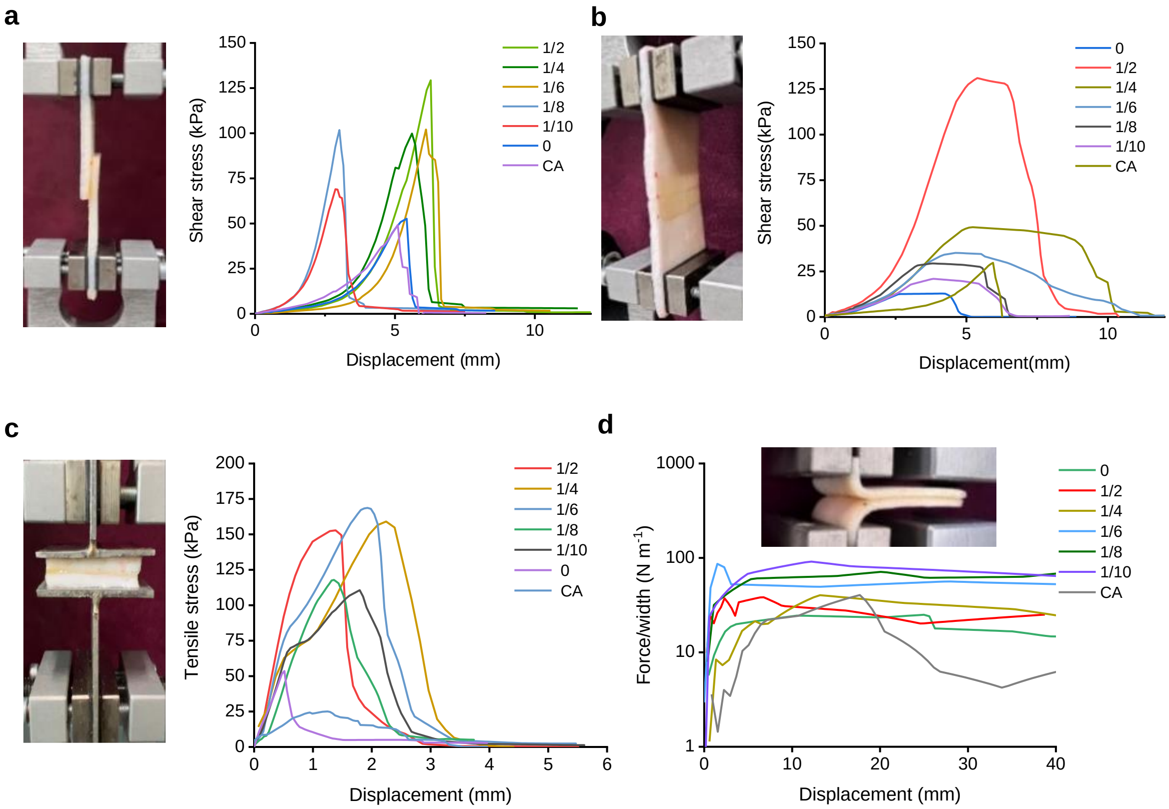


**Supplementary Fig. 20 | Representative photos and test curves of the BDRAs on wet soft tissue. a**, **b**, **c**, **d**，Shear test (**a**), wound closure strength test (**b**), tensile test (**c**), and T-Peel test (**d**) between wet porcine skins and the BDRAs (MDO-HEA-NHS). The molar ratio of MDO to HEA was 1:1, and NHS:MDO (mol:mol) was from 0 to 1/2. CA (2-octyl cyanoacrylate) was chosen as the control.

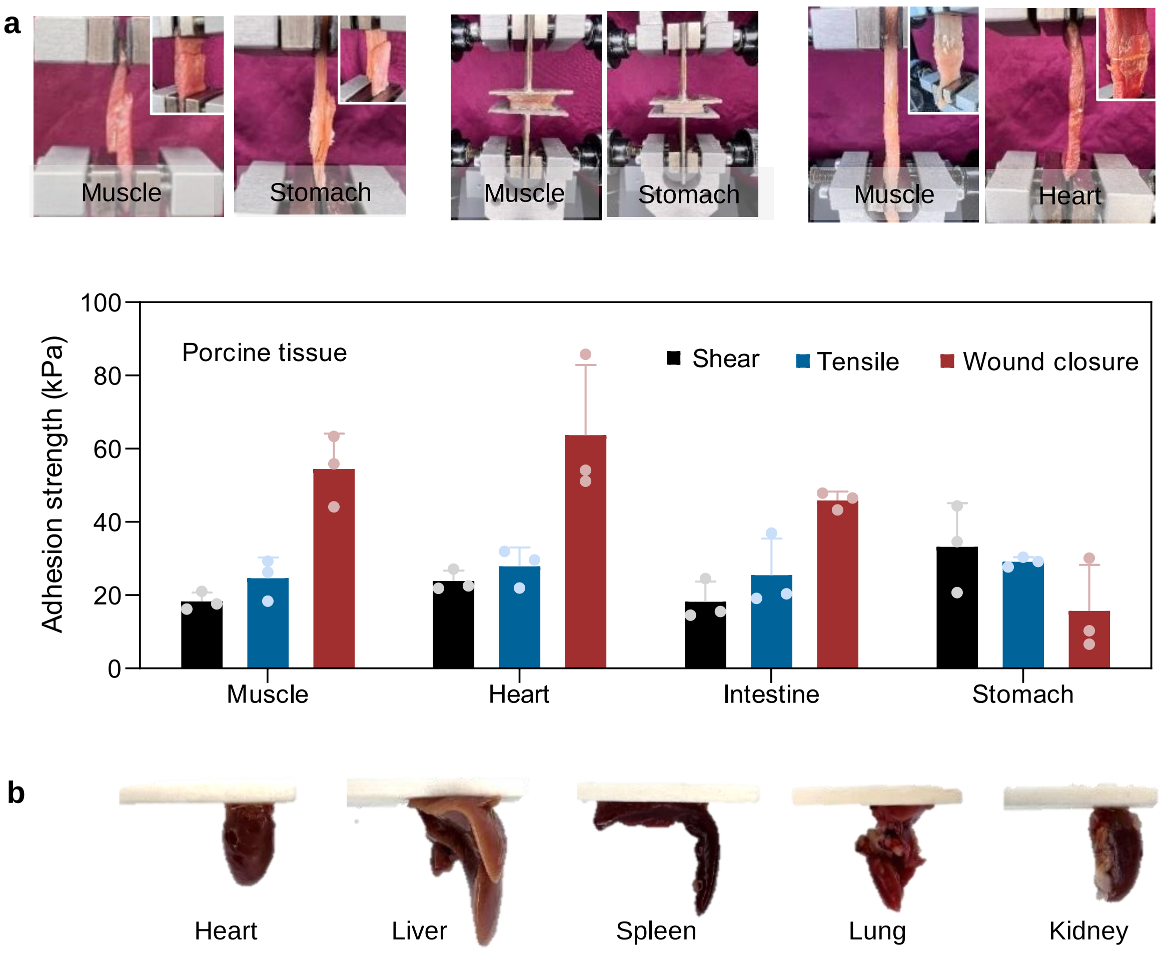


**Supplementary Fig. 21 | Adhesion performance between various tissues adhered by the BDRA. a**, Shear, tensile and wound closure adhesion strength tests for various organs adhered by the BDRA ($MDO_1$-$HEA_1$-$NHS_{1/2}$). Data are presented as means ± SDs for $n$ = 3 independent samples. **b**, Representative photos of BDRA ($MDO_1$-$HEA_1$-$NHS_{1/2}$) with various biological organs.

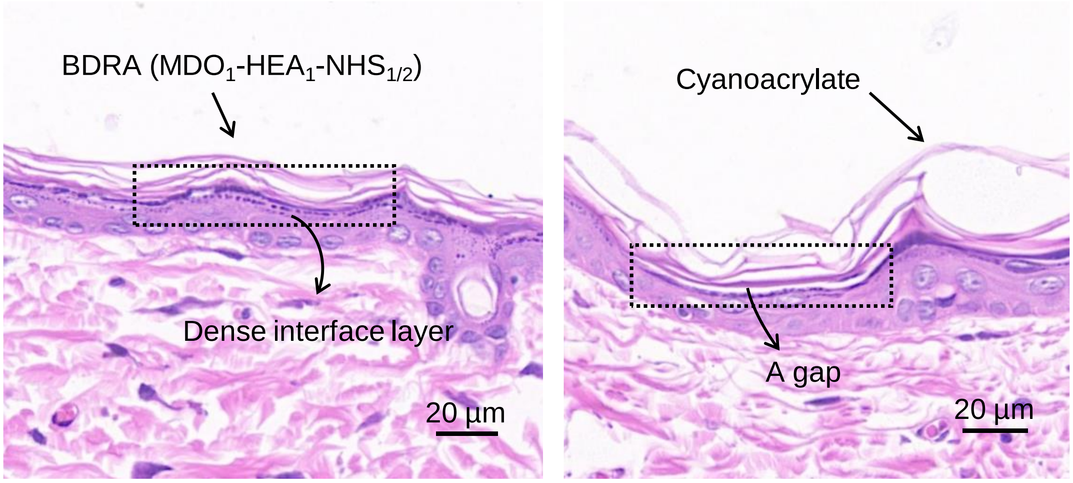


**Supplementary Fig. 22 | Adhesive interface between soft tissue skin and the BDRA.** Representative H&E staining of the skin after Vetbond (N-butyl cyanoacrylate) and the BDRA ($MDO_1$-$HEA_1$-$NHS_{1/2}$) treatment. Scale bar, 20 μm. Three times each experiment were repeated independently with similar results.

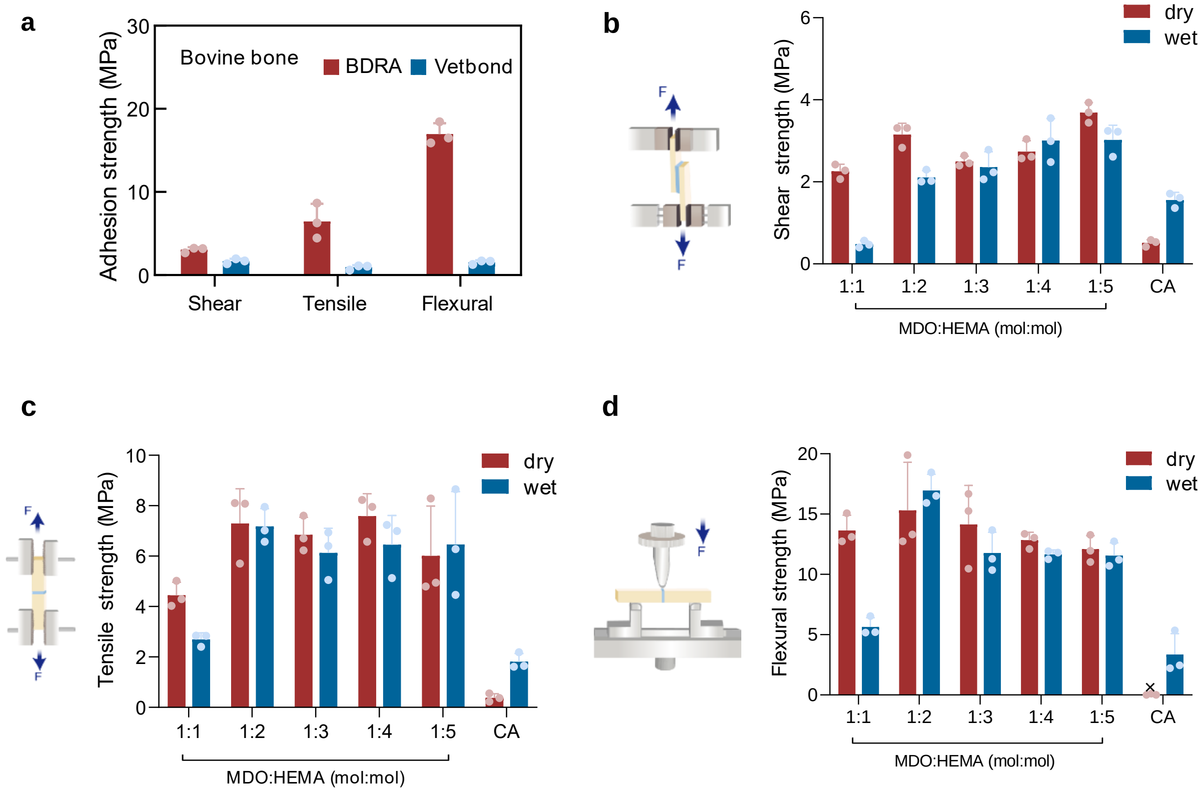


**Supplementary Fig. 23 | Characterization on adhesive strength of the BDRAs via ex vivo bovine bone samples. a**, Adhesion performance of the adaptive BDRA ($MDO_1$-$HEMA_2$) for hard tissues. The *N*-butyl cyanoacrylate (Vetbond) was chosen as a reference. **b**, **c**, **d**, The shear (**b**) and tensile (**c**) and flexural adhesion strength (**d**) of the BDRAs (MDO-HEMA) with varying the molar ratio of MDO to HEMA from 1:1 to 1:5. Data are presented as means ± SDs for $n = 3$ independent samples.

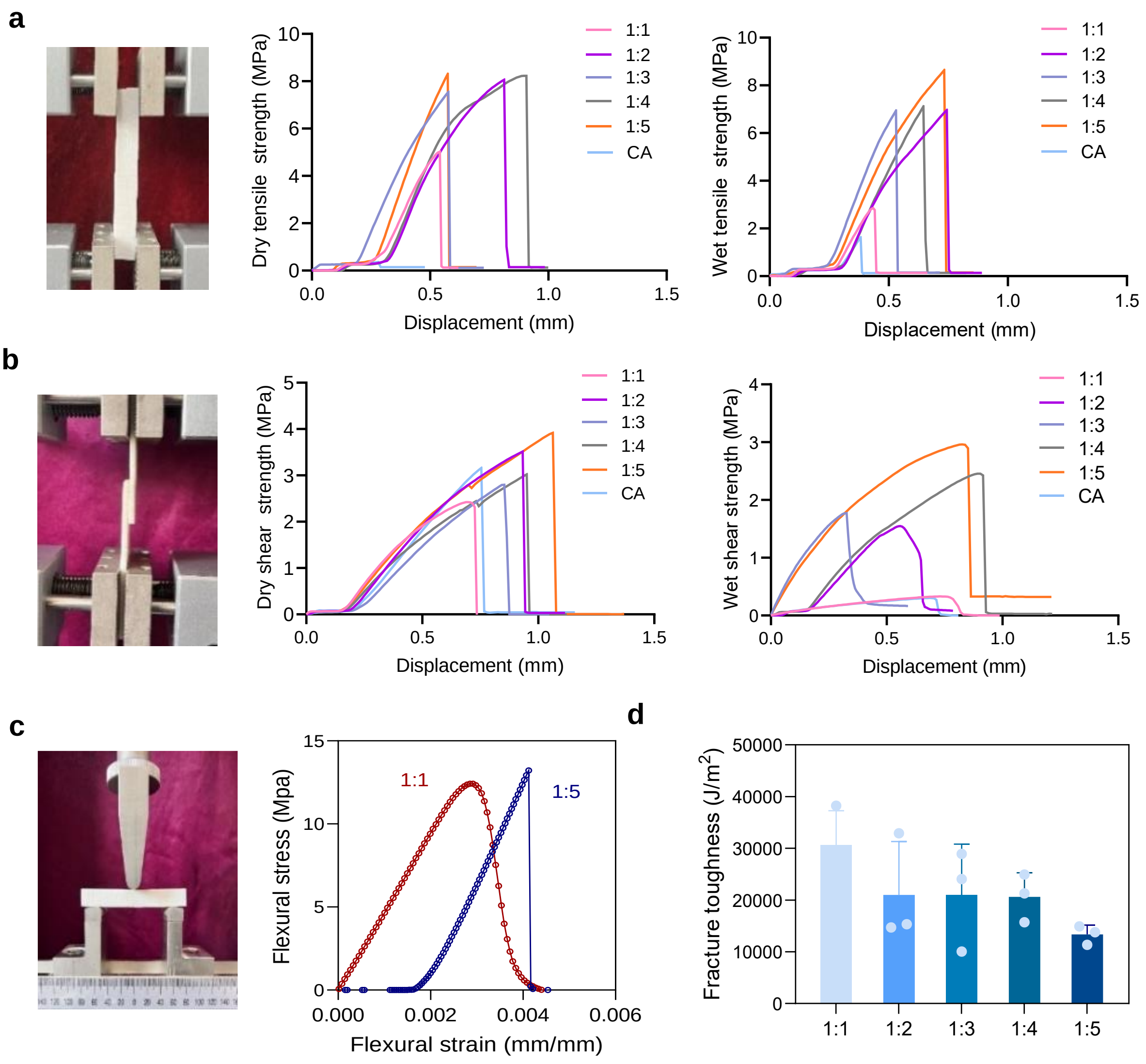


**Supplementary Fig. 24 | Representative photos and test curves of the BDRAs for dry and wet bovine bones. a**, **b**, **c**, The tensile test (**a**), shear test (**b**), and 3-point bend test (**c**) for the BDRAs (MDO-HEMA, with the varying molar ratio of MDO to HEMA from 1:1 to 1:5) adhesion. **d**, Fracture toughness of the BDRAs (MDO-HEMA). Data are presented as means ± SDs for $n$ = 3 independent samples.

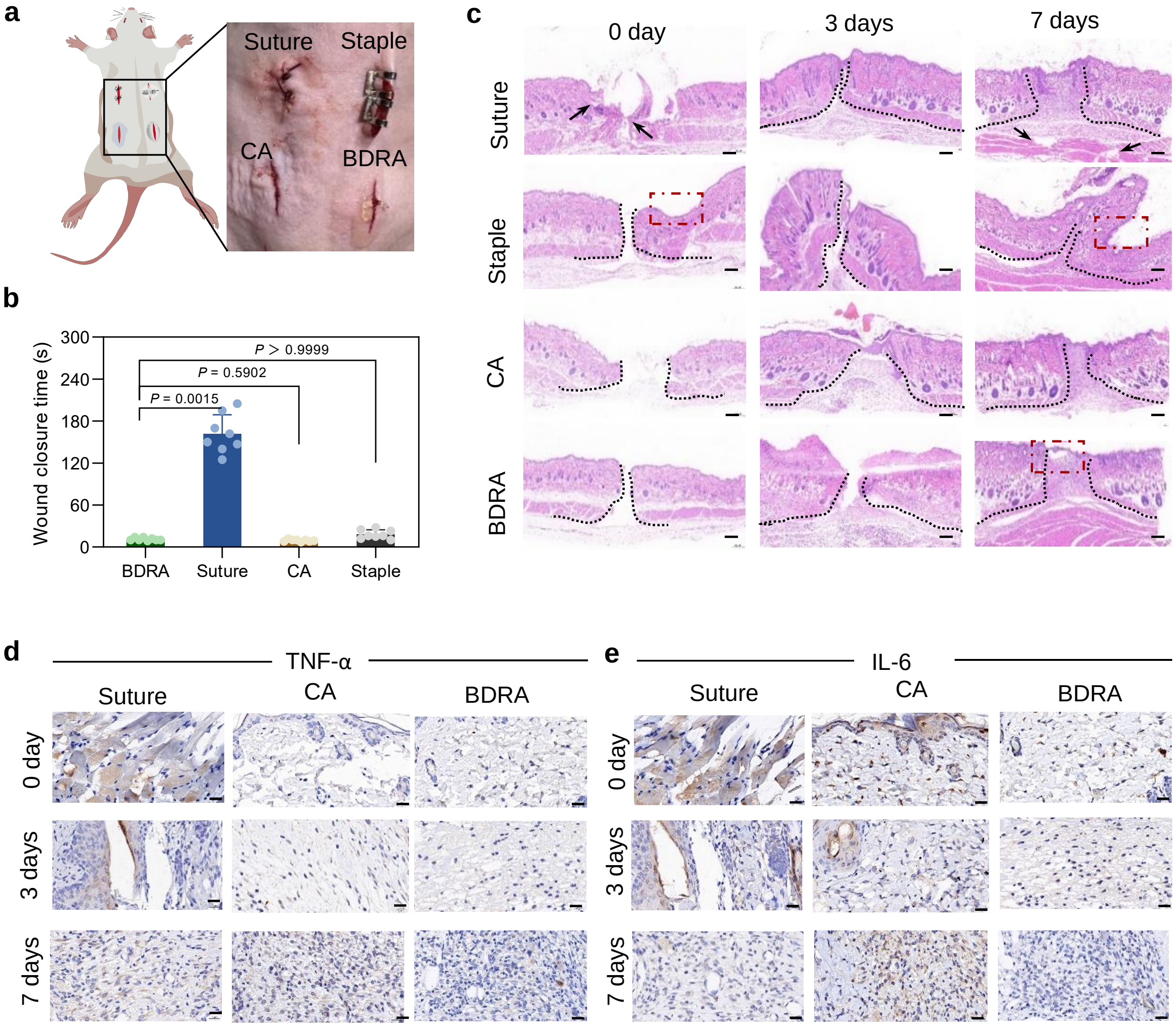


**Supplementary Fig. 25 | Linear wound closure on rat skin. a**, Schematic diagram and photograph of suture, staple, CA, and the BDRA ($MDO_1$-$HEA_1$-$NHS_{1/2}$) treatment on the back of rats. **b**, Quantitative analysis for implementation time of wound closure treatment, data are presented as means ± SDs, *n* = 8 independent samples. One-way analyses of variance (ANOVA) were used for the statistical analysis. **c**, H&E staining after treatment of linear wound for 3 and 7 days, scale bar = 200 μm. **d, e,** Immumohistochemical staining of tumor necrosis factor-α (TNF-α) (**d**) and proinflammatory cytokine IL-6 (**e**) in damaged skin tissues. Scale bars are 20 μm. The experiments in (**c-e**) were repeated independently three times with similar results.

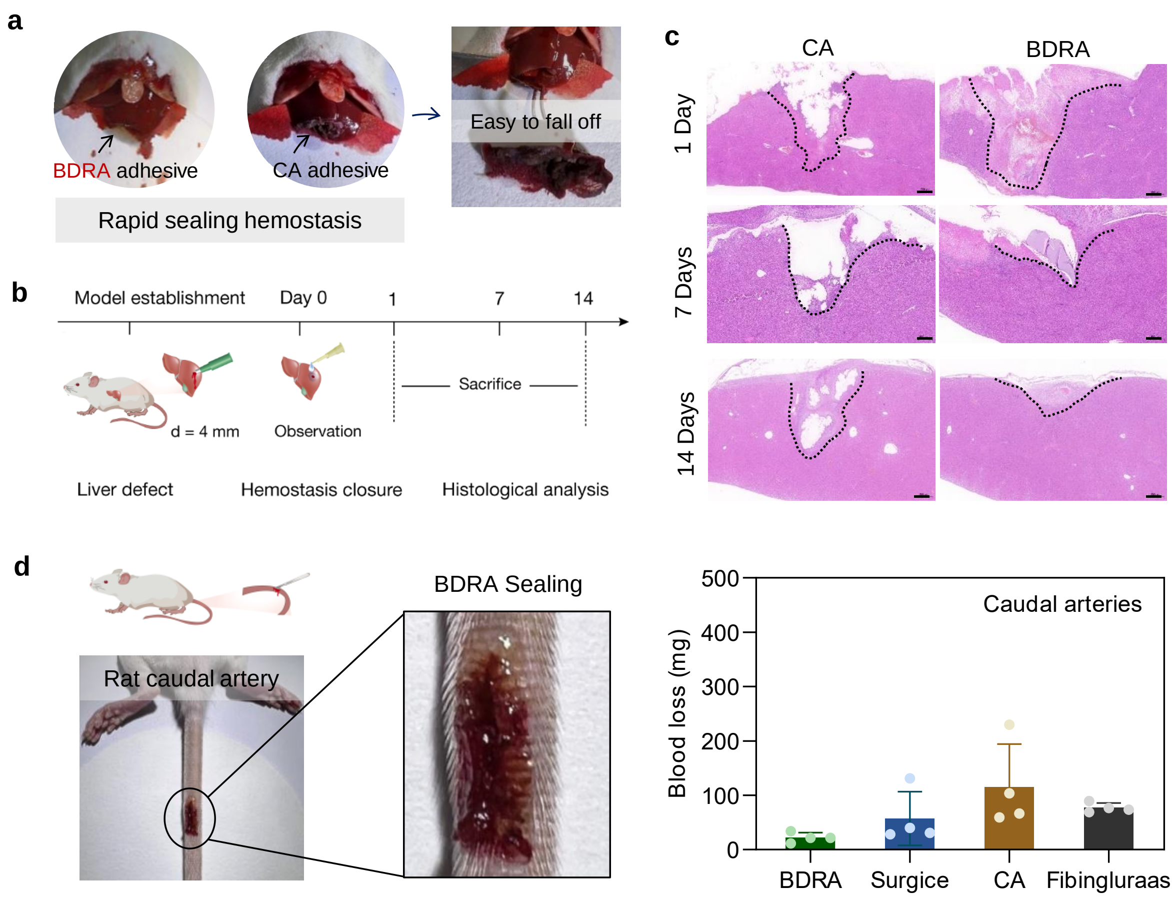


**Supplementary Fig. 26 | Hemostasis performances. a**, Representative images for hemostasis of liver amputation injury treated with the BDRA ($MDO_1$-$HEA_1$-$NHS_{1/2}$) and CA glue. Data are presented as means ± SDs, $n = 4$ independent samples. **b**, Illustration of liver perforation model of rat. **c**, Representative H&E images of the damaged rat liver after sealing by BDRA ($MDO_1$-$HEA_1$-$NHS_{1/2}$) or CA (Vetbond) for 1, 7, 14 days. Scale bars are 500 μm. Three times each experiment were repeated independently with similar results. **d**, The blood loss of lacerated rat caudal arteries.

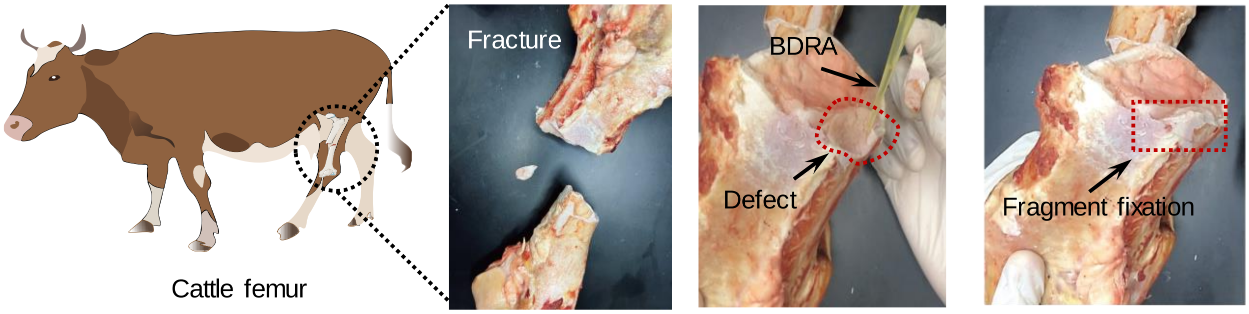


**Supplementary Fig. 27 |** Adhesion of BDRA ($MDO_1$-$HEMA_1$) on an ex vivo small fragment of bovine bone.

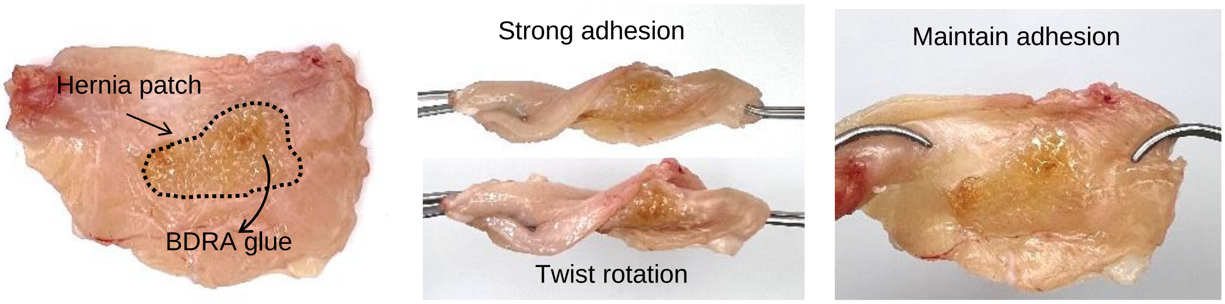


**Supplementary Fig. 28 |** Polypropylene hernia patch adhered to fresh abdominal muscles by the BDRA ($MDO_1$-$HEA_1$-$NHS_{1/2}$).

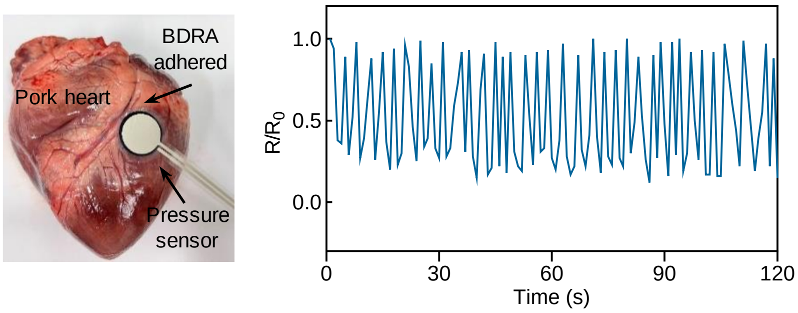


**Supplementary Fig. 29 |** Adhesion of a pressure sensor on ex vivo porcine heart by a BDRA ($MDO_1$-$HEA_1$-$NHS_{1/2}$) and the relative resistance variations of the BDRA adhered sensor under intermittently pressure.

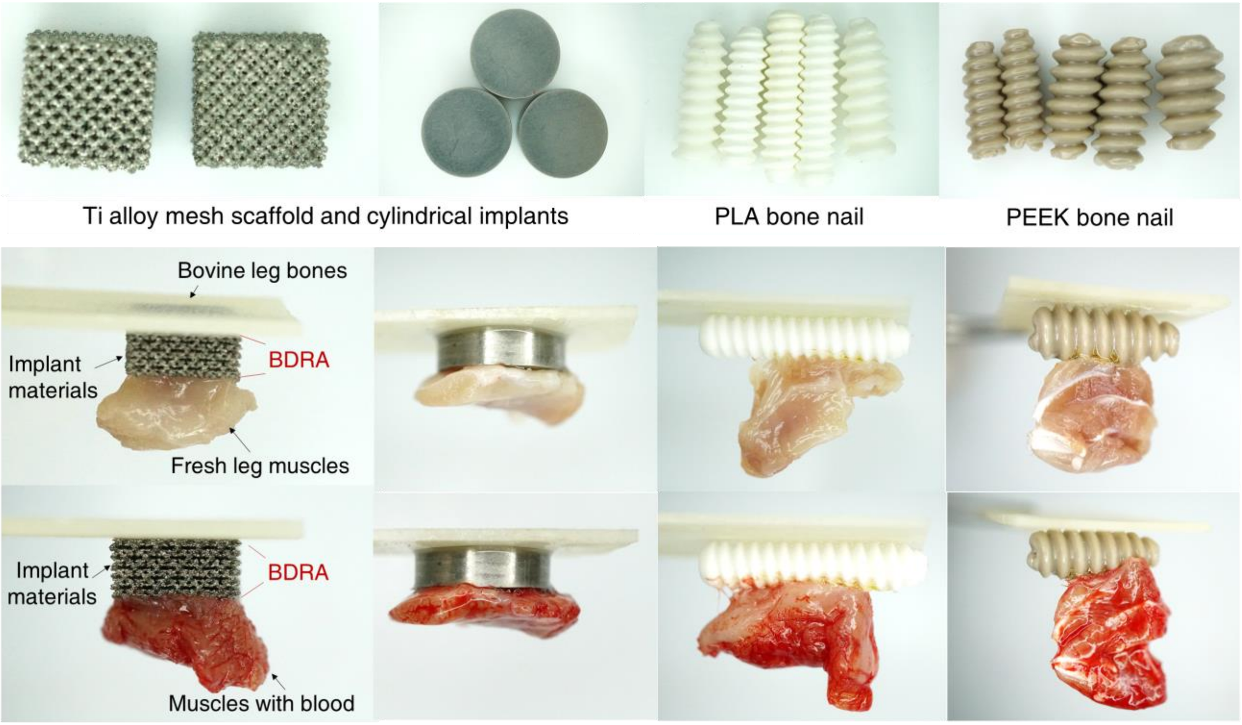


**Supplementary Fig. 30 |** Adhesion of clinical orthopedic materials with different shapes adhered to bone and muscle by the BDRA ($MDO_1$-$HEMA_1$).

## Supplementary Table

**Supplementary Table 1 |** Comparison of degradability, setting time and adhesion strength of the existing tissue adhesives for hard and soft tissues.

| **Soft tisusse** | | | | **Hard tisusse** | | | |
|---|---|---|---|---|---|---|---|
| **Degradability** | **Time** | **Adhesive strength** | **ref** | **Degradability** | **Time** | **Adhesive strength** | **ref** |
| √ | 0.1-30 min | 115-182 kPa | This work | √ | 0.1-30 min | 3-18 MPa | This work |
| √ | 40 min | 151 kPa | (*26*) | √ | 2.5 min | 108 kPa | (*37*) |
| √ | 5 min | 3-7 kPa | (*49*) | √ | 2 min | 49 kPa | (*14*) |
| × | < 3 s | 15-20 kPa | (*44*) | √ | 100 s | 770 kPa | (*21*) |
| × | 3 min | 75 kPa | (*28*) | √ | 5-20 min | 10-20 kPa | (*17*) |
| × | < 3 s | 28 kPa | (*41*) | √ | 20 s | 30-50 kPa | (*11*) |
| × | 14 s | 23 kPa | (*47)* | √ | 10 min | 1-2 MPa | (*16*) |
| √ | 30 s | 20-40 kPa | (*12*) | × | 50 s | 9 MPa | (*10*) |
| √ | 240 s | 90 kPa | (*32*) | | | | |
| √ | 10 s | 84 kPa | (*19*) | | | | |

**Supplementary Table 2 |** Setting time of MDO copolymerizing with eighteen comonomers.

| Comonomer name | CAS number | Setting time (t) | Structural formula | Classification |
|---|---|---|---|---|
| **2-Hydroxyethyl acrylate (HEA)** | 818-61-1 | 5 s | 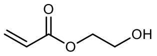 | |
| **Acrylic acid (AA)** | 79-10-7 | 9 s | 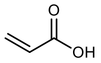 | **(t<30 s)** |
| **Hydroxypropyl acrylate (HPA)** | 25584-83-2 | 12 s | 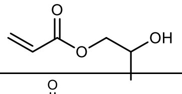 | |
| **3-chloro-2-hydroxypropyl methacrylate (HPMA-Cl)** | 13159-52-9 | 31 s | 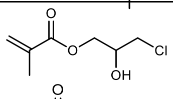 | |
| **Methyl acrylate (MA)** | 96-33-3 | 32 s | 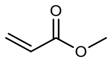 | |
| **Ethyl acrylate (EA)** | 140-88-5 | 45 s | 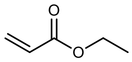 | **(t<60 s)** |
| **Poly (ethylene glycol) methacrylate (PEGMA)** | 25736-86-1 | 55 s | 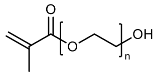 | |
| **2-Hydroxyethyl methacrylate (HEMA)** | 868-77-9 | 58 s | 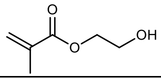 | |
| **Butyl Acrylate (BA)** | 141-32-2 | 60 s | 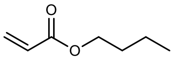 | |
| **Methacrylic acid (MAA)** | 79-41-4 | 64 s | 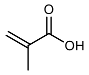 | **(1<t<3 min)** |
| **2-Ethylhexyl acrylate (2-EHA)** | 103-11-7 | 2 min | 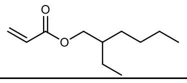 | |
| **2-Isocyanatoethyl methacrylate (IEM)** | 30674-80-7 | 7 min | 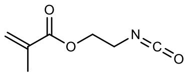 | |
| **Benzyl methacrylate (BzMA)** | 2495-37-6 | 12 min | 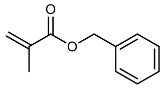 | |
| **3-(Trimethoxysilyl)propyl methacrylate (TMSPMA)** | 2530-85-0 | 13 min | 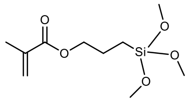 | **(3<t<30 min)** |
| **2,2,2-Trifluoroethyl methacrylate (TFEMA)** | 352-87-4 | 20 min | 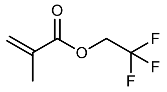 | |
| **2-Ethylhexyl methacrylate (2-EHMA)** | 688-84-6 | 28 min | 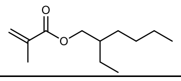 | |
| **Glycidyl methacylate (GMA)** | 106-91-2 | 31 min | 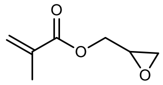 | **( t>30 min )** |
| **Methyl methacrylate (MMA)** | 80-62-6 | 40 min | 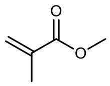 | |
