# Supplementary material for: Tunable backbone-degradable robust tissue adhesives via in situ radical ring-opening polymerization
Source: Nat Commun. 2023 Sep 28;14:6063. doi: 10.1038/s41467-023-41610-1 (PMC10539349; doi:10.1038/s41467-023-41610-1)
Supplement: Supplementary file 2 — Reporting Summary [file 41467_2023_41610_MOESM2_ESM.pdf]

nature portfolio

Corresponding author(s): Binggang Chen,Qiuyan Yan,Shifang Luan

Last updated by author(s): Sep 7, 2023

Reporting Summary

Nature Portfolio wishes to improve the reproducibility of the work that we publish. This form provides structure for consistency and transparency in reporting. For further information on Nature Portfolio policies, see our Editorial Policies and the Editorial Policy Checklist.

Statistics

For all statistical analyses, confirm that the following items are present in the figure legend, table legend, main text, or Methods section.

n/a	Confirmed	
☐	☒	The exact sample size (*n*) for each experimental group/condition, given as a discrete number and unit of measurement
☐	☒	A statement on whether measurements were taken from distinct samples or whether the same sample was measured repeatedly
☐	☒	The statistical test(s) used AND whether they are one- or two-sided<br>*Only common tests should be described solely by name; describe more complex techniques in the Methods section.*
☒	☐	A description of all covariates tested
☒	☐	A description of any assumptions or corrections, such as tests of normality and adjustment for multiple comparisons
☐	☒	A full description of the statistical parameters including central tendency (e.g. means) or other basic estimates (e.g. regression coefficient) AND variation (e.g. standard deviation) or associated estimates of uncertainty (e.g. confidence intervals)
☐	☒	For null hypothesis testing, the test statistic (e.g. *F*, *t*, *r*) with confidence intervals, effect sizes, degrees of freedom and *P* value noted<br>*Give P values as exact values whenever suitable.*
☒	☐	For Bayesian analysis, information on the choice of priors and Markov chain Monte Carlo settings
☒	☐	For hierarchical and complex designs, identification of the appropriate level for tests and full reporting of outcomes
☒	☐	Estimates of effect sizes (e.g. Cohen's *d*, Pearson's *r*), indicating how they were calculated

Our web collection on statistics for biologists contains articles on many of the points above.

Software and code

Policy information about availability of computer code

Data collection	1H NMR spectra were recorded on a Bruker AVANCE III 500 MHz spectrometer.<br>GPC data were recorded using a PL-GPC 120 Integrated GPC System.<br>Water contact angle were performed on DSA100 drop-shape analyzer (KRÜSS, Hamburg, Germany).<br>Mechanical and adhesion properties were tested by LR10K Plus universal testing machine (AMETEK-Lloyd, USA) and AGS-X universal testing machine (SHIMADZU, Kyoto, Japan) .<br>The glass transition temperature was recorded by a DMA 850 analyzer (New Castle, USA) .<br>The CT images were collected by a Bruker microCT SkyScan 1172.<br>The stained cells were observed by Zeiss Confocal LSM 700.
Data analysis	Digital microscopic images were analyzed by using Case Viewer (Version: 2.4.0.119028). 1H NMR peak fitting was implemented on software MestReNova-14.1.2. All statistical analyses have conducted by using Graph Pad Prism (version: 9.0.0). CT images were reconstructed and analyzed by Bruke DataViewer (1.5.1.9), CTvox (3.0) and CT-Analyser (1.15.4.0).

For manuscripts utilizing custom algorithms or software that are central to the research but not yet described in published literature, software must be made available to editors and reviewers. We strongly encourage code deposition in a community repository (e.g. GitHub). See the Nature Portfolio guidelines for submitting code & software for further information.

Data

Policy information about availability of data

All manuscripts must include a data availability statement. This statement should provide the following information, where applicable:

- Accession codes, unique identifiers, or web links for publicly available datasets
- A description of any restrictions on data availability
- For clinical datasets or third party data, please ensure that the statement adheres to our policy

All data are available in the main text, Supplementary Information, or Source Data file. Source data are provided with this paper. If any raw data files are needed in another format, they are available from the corresponding author upon request.

Research involving human participants, their data, or biological material

Policy information about studies with human participants or human data. See also policy information about sex, gender (identity/presentation), and sexual orientation and race, ethnicity and racism.

Reporting on sex and gender	N/A
Reporting on race, ethnicity, or other socially relevant groupings	N/A
Population characteristics	N/A
Recruitment	N/A
Ethics oversight	N/A

Note that full information on the approval of the study protocol must also be provided in the manuscript.

Field-specific reporting

Please select the one below that is the best fit for your research. If you are not sure, read the appropriate sections before making your selection.

☒ Life sciences ☐ Behavioural & social sciences ☐ Ecological, evolutionary & environmental sciences

For a reference copy of the document with all sections, see nature.com/documents/nr-reporting-summary-flat.pdf

Life sciences study design

All studies must disclose on these points even when the disclosure is negative.

Sample size	No statistical method was used to predetermine the sample size for each study. The appropriate sample size was used based on the published literatures on similar evaluations (such as Nature 575, 169-174 (2019) doi: 10.1038/ s41586-019-1710-5). For in vitro studies,the appropriate sample size (n=3-6) was used for ex vivo experiments. For in vivo studies, each group contains 3-4 for evaluating the statistical significance. A precise value of 'n' were provided in the legends of figures. Sample size was chosen to ensure reproducibility of the experiments in accordance with the replacement, reduction and refinement principles of animal ethics regulation.
Data exclusions	No data was excluded from the analyses.
Replication	All experiments were performed with independent replicates. At least three independent samples were performed for each experiment.
Randomization	All samples were randomly allocated into experimental groups.
Blinding	The operator was blinded to group allocation during H&E staining. Blinding was not relevant to other work since the metrics were quantified and objectively analyzed.

Reporting for specific materials, systems and methods

We require information from authors about some types of materials, experimental systems and methods used in many studies. Here, indicate whether each material, system or method listed is relevant to your study. If you are not sure if a list item applies to your research, read the appropriate section before selecting a response.

Materials & experimental systems

n/a	Involved in the study
☒	☐ Antibodies
☐	☒ Eukaryotic cell lines
☒	☐ Palaeontology and archaeology
☐	☒ Animals and other organisms
☒	☐ Clinical data
☒	☐ Dual use research of concern
☒	☐ Plants

Methods

n/a	Involved in the study
☒	☐ ChIP-seq
☒	☐ Flow cytometry
☒	☐ MRI-based neuroimaging

Eukaryotic cell lines

Policy information about cell lines and Sex and Gender in Research

Cell line source(s)	In this study, the L929 murine fibroblast cell line (FH0534) and murine osteoblast precursor cells MC3T3-E1 (FH0382) were purchased from FuHeng Biology (Shanghai, China).
Authentication	L929 and MC3T3-E1 cells from FuHeng Biology Co., Ltd. were authenticated by FuHeng based on STR profiling technology.
Mycoplasma contamination	The cell lines were not additionally validated in this study. The cell lines were tested negative for mycoplasma contamination when they came out of the library.
Commonly misidentified lines (See ICLAC register)	No commonly misidentified cell lines were used in the study.

Animals and other research organisms

Policy information about studies involving animals; ARRIVE guidelines recommended for reporting animal research, and Sex and Gender in Research

Laboratory animals	Female Sprague-Dawley rats (SD rats, 100-120 g, 4-5 weeks old or 140-160 g, 5-6 weeks old) were used for our experiments, and were housed at an ambient temperature of 25 °C (24–26 °C) and humidity of 30 %, and allowed access to a standard diet and water ad libitum. Female BALB/c mice (18-20 g, 8-9 weeks old) were used for pulmonary toxicity test, and replenishing water and nutrients with fresh apples.
Wild animals	This study did not involve wild animals.
Reporting on sex	All female animals were used in this study, and gender differences were not considered. The findings were supposed to apply to both female and male rat because the result should be sex-independent.
Field-collected samples	This study did not involve sample collected from the field.
Ethics oversight	All animal experiments were performed in accordance with the Guidelines for the Care and Use of Laboratory Animals of the Chinese Academy of Sciences and approved by the Animal Ethics Committee of Changchun institute of Applied Chemistry, Chinese Academy of Sciences (no. 20220003).

Note that full information on the approval of the study protocol must also be provided in the manuscript.
