# Supplementary material for: Tunable backbone-degradable robust tissue adhesives via in situ radical ring-opening polymerization
Source: Nat Commun. 2023 Sep 28;14:6063. doi: 10.1038/s41467-023-41610-1 (PMC10539349; doi:10.1038/s41467-023-41610-1)
Supplement: Supplementary file 3 — Description of Additional Supplementary Files [file 41467_2023_41610_MOESM3_ESM.pdf]

Title: Supplementary Movie 1:
Description: A fractured bovine bone bonded by the BDRA bears a weight of 60 kg.

Title: Supplementary Movie 2:
Description: The wound closure test of the BDRA to pigskin.

Title: Supplementary Movie 3:
Description: Wound closure treatment by BDRA in a rat dorsal skin model.

Title: Supplementary Movie 4:
Description: The rapid sealing hemostasis of the BDRAs for liver and carotid injuries.

Title: Supplementary Movie 5:
Description: Skull fragment repositioned by the BDRA
